# An Update on Sec61 Channel Functions, Mechanisms, and Related Diseases

**DOI:** 10.3389/fphys.2017.00887

**Published:** 2017-11-01

**Authors:** Sven Lang, Stefan Pfeffer, Po-Hsien Lee, Adolfo Cavalié, Volkhard Helms, Friedrich Förster, Richard Zimmermann

**Affiliations:** ^1^Competence Center for Molecular Medicine, Saarland University Medical School, Homburg, Germany; ^2^Department of Molecular Structural Biology, Max-Planck Institute of Biochemistry, Martinsried, Germany; ^3^Center for Bioinformatics, Saarland University, Saarbrücken, Germany; ^4^Experimental and Clinical Pharmacology and Toxicology, Saarland University, Homburg, Germany; ^5^Bijvoet Center for Biomolecular Research, Utrecht University, Utrecht, Netherlands

**Keywords:** ATP import, BiP, calcium leakage, endoplasmic reticulum, protein biogenesis, Sec61 complex

## Abstract

The membrane of the endoplasmic reticulum (ER) of nucleated human cells harbors the protein translocon, which facilitates membrane integration or translocation of almost every newly synthesized polypeptide targeted to organelles of the endo- and exocytotic pathway. The translocon comprises the polypeptide-conducting Sec61 channel and several additional proteins and complexes that are permanently or transiently associated with the heterotrimeric Sec61 complex. This ensemble of proteins facilitates ER targeting of precursor polypeptides, modification of precursor polypeptides in transit through the Sec61 complex, and Sec61 channel gating, i.e., dynamic regulation of the pore forming subunit to mediate precursor transport and calcium efflux. Recently, cryoelectron tomography of translocons in native ER membrane vesicles, derived from human cell lines or patient fibroblasts, and even intact cells has given unprecedented insights into the architecture and dynamics of the native translocon and the Sec61 channel. These structural data are discussed in light of different Sec61 channel activities including ribosome receptor function, membrane insertion, and translocation of newly synthesized polypeptides as well as the putative physiological roles of the Sec61 channel as a passive ER calcium leak channel. Furthermore, the structural insights into the Sec61 channel are incorporated into an overview and update on Sec61 channel-related diseases—the Sec61 channelopathies—and novel therapeutic concepts for their treatment.

## Introduction

**Figure 1:**
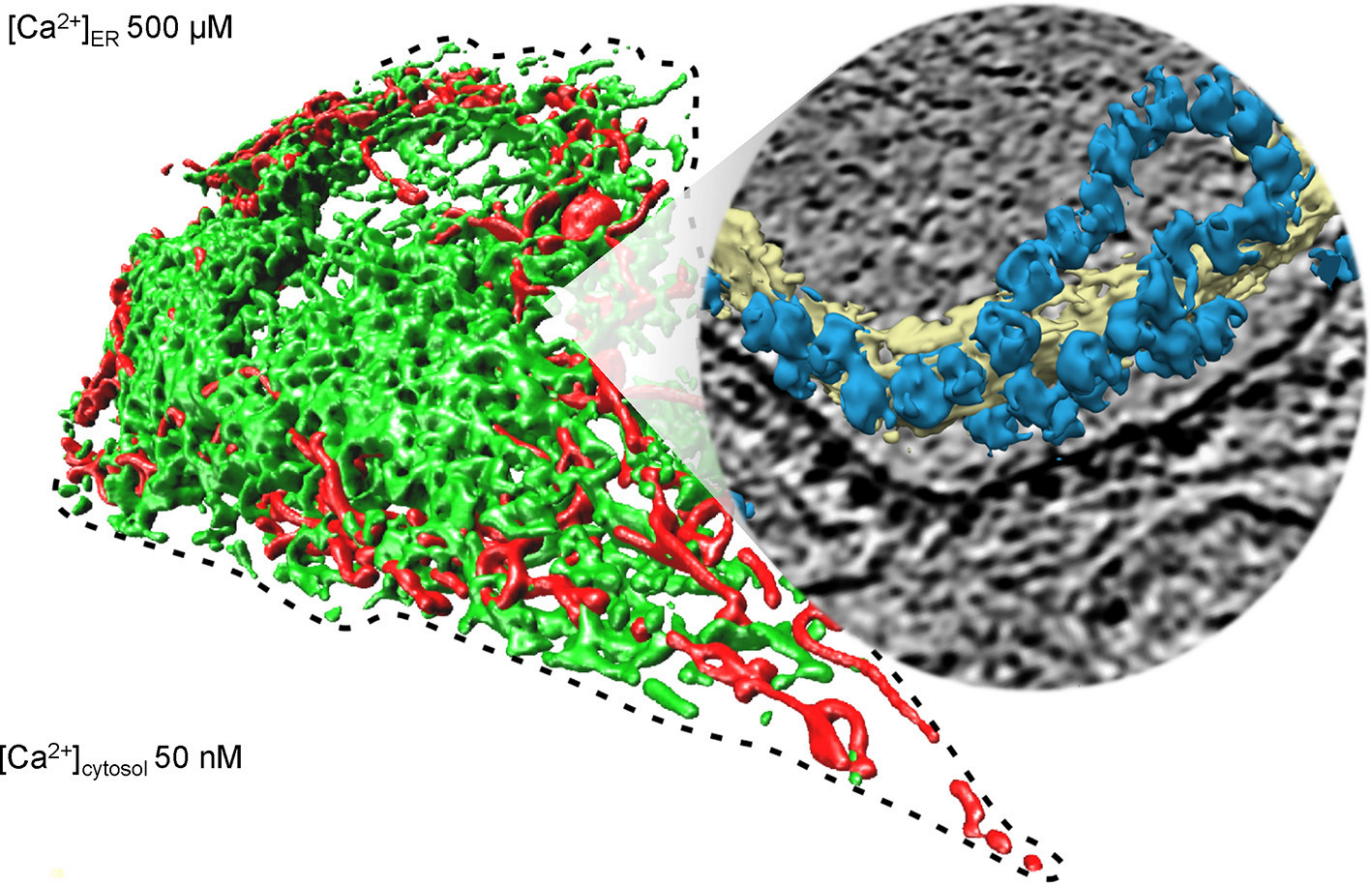
Collage of 3D reconstructions of mammalian mitochondria and ER, respectively. The left part of the figure represents a 3D reconstruction after live cell fluorescence imaging, following import of a green fluorescent protein into the ER and of a red fluorescent protein into the mitochondria. The plasma membrane is indicated by a dashed line; the position of the round nucleus can be estimated in the upper part of the cell void of ER and mitochondria. Typical concentrations of free calcium are given for cytosol and ER of a resting cell. The right part represents a 3D reconstruction of cellular ER after CET, on top of a slice through the respective tomogram. ER membranes are shown in yellow; 80S ribosomes are shown in blue. The collage is based on Zimmermann (2016).

The endoplasmic reticulum (ER) represents the largest continuous tubular membrane network within nucleated mammalian cells (Friedman and Voeltz, 2011; Figure 1). Its striking dynamics were recently demonstrated via lattice light-sheet microscopy (Valm et al., 2017). While occupying up to a third of a cell's volume at any given time, the ER managed to “scan” and explore over 97% of a cell's volume within 15 min. Not surprisingly, this high mobility allows the ER to be the organelle with the highest contact rate to other compartments of the endomembrane system, such as lipid droplets or mitochondria and, therefore, the nexus of inter-organelle tethering. Together with the size of the ER comes both an array of different functions and morphological structures. The former include lipid and steroid synthesis, calcium storage, protein transport, maturation, and proteostasis some of which are assumed to occur at distinct ER subdomains (Blobel and Dobberstein, 1975; Palade, 1975; Berridge, 2002; Brostrom and Brostrom, 2003; Clapham, 2007; Braakman and Bulleid, 2011). The latter include the nuclear envelope and the peripheral ER consisting of smooth tubular and rough sheet-like areas. Recently, advances in super-resolution imaging of live and fixed cells extended the concept of tubular and sheet-like peripheral ER domains by introducing ER matrices, densely packed ER tubular arrays, to the portfolio of ER structural domains. The combination of nanoscopic approaches revealed two features. One, the peripheral ER moves at high speeds broadly dependent on cellular energy sources. And two, many of the peripheral ER structures classically identified as sheets represent instead dense matrices of convoluted tubules (Nixon-Abell et al., 2016). In the context of the ER, rough and smooth refers to the presence or absence of membrane-associated ribosomes or polysomes on the cytosolic surface. The density of bound ribosomes is considered one driver for the formation of sheets. However, common to tubes, matrices and sheets is the lumenal distance of about 50 nm in mammalian cells most likely established by lumenal spacer proteins such as Climp-63 (Shibata et al., 2006, 2010; Schwarz and Blower, 2016). Furthermore, advances in ultrathin sectioning of electron microscopy preparations visualize ER sheets, especially juxtanuclear ones, being stacked in a parking garage like fashion with interconnecting helicoidal ramps to allow dense packing in a crowded environment of neuronal and secretory salivary gland cells (Terasaki et al., 2013; Nixon-Abell et al., 2016).

**Figure 2:**
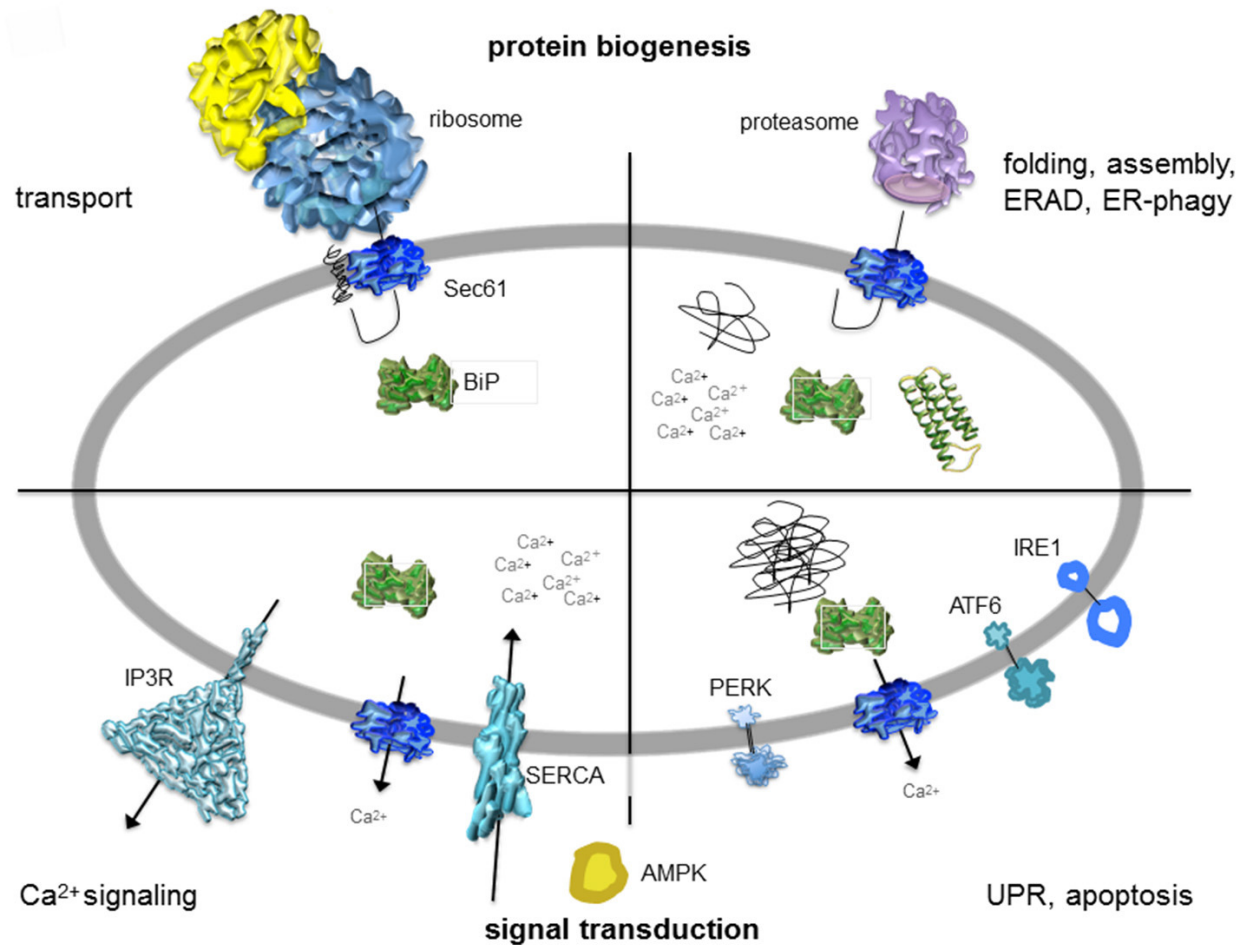
Artist's depiction of cross-section through the mammalian ER with a focus on signal transduction and protein biogenesis. The non-annotated structures refer to a not yet-folded polypeptide, a natively folded protein, and an aggregate of non-native polypeptides, respectively. AMPK, AMP-activated protein kinase; IP3R, IP3-receptor; SERCA, sarcoplasmic/endoplasmic reticulum Ca^2+^ ATPase. The cartoon is based on Zimmermann (2016). See text for details.

The heterotrimeric Sec61 complex in the ER membrane provides the dynamic polypeptide-conducting channel, which mediates membrane insertion of most membrane proteins of organelles involved in endo- and exocytosis and translocation of all precursors of polypeptides destined for these same organelles and most precursors of secretory proteins (Görlich et al., 1992) (“transport” in Figure 2). With respect to membrane proteins, the exceptions are tail-anchored (TA) membrane proteins (reviewed by Rabu et al., 2009; Borgese and Fasana, 2011), and with respect to secretory proteins, the mechanistically completely unrelated “unconventional secretion” is the alternative mechanism and described in detail elsewhere (Nickel and Rabouille, 2009). Precursors of soluble polypeptides and membrane proteins are targeted to the Sec61 complex via their amino-terminal signal peptides or transmembrane helices either during their synthesis (termed cotranslationally) or after completion of their synthesis (termed posttranslationally) (Blobel and Dobberstein, 1975; von Heijne, 1986). Predominantly, cotranslational targeting is supposed to involve the cytosolic signal recognition particle (SRP) plus its receptor on the ER surface, SRP receptor (SR) (Table 1); posttranslational targeting can involve one of several SRP-independent targeting machineries, which typically also comprise cytosolic and ER membrane resident components and may also interact with ribosomes. Thus, there is substrate overlap and redundancy in these targeting machineries that we are only beginning to appreciate. This is described in more detail below, under the subheading “Targeting of Precursor Polypeptides to the Sec61 Complex in the Human ER membrane.”

**Table 1:** Protein transport components and associated proteins in HeLa cells.

**Component/ - Subunit**	**Abundance**	**Location**	**Linked diseases**
Calmodulin	9,428	C	
**Cytosolic Chaperones**		C	
- Hsc70 (HSPA8)	3,559		
- Hdj2 (DNAJA1)			
- Bag1 (HAP, RAP46)			
**#NAC**		C	
- NACα	1,412		
- NACβ			
**#SRP**		C	
- SRP72	355		
- SRP68	197		
- SRP54	228		
- SRP19	33		
- SRP14	4,295		
- SRP9	3,436		
- 7SL RNA			
**SRP receptor**		ERM	
- SRα (docking protein)	249		
- SRβ	173		
- hSnd1	?		
**Snd receptor**			
- hSnd2 (TMEM208)	81	ERM	
- hSnd3	?		
**#Bag6 complex**		C	
- TRC35			
- Ubl4A			
- Bag6 (Bat3)			
SGTA		C	
TRC40 (Asna-1)		C	
**TA receptor**		ERM	
- CAML	5		Down syndrome, Congenital heart disease
- WRB (CHD5)	4		
- #Sec62 (TLOC1)	26	ERM	Prostate cancer, Lung cancer
**#Sec61 complex**		ERM	
- Sec61α1	139		Diabetes, Common Variable Immune Deficiency (CVID), Tubulo-interstitial kidney disease (TKD)
- Sec61β	456		Polycystic Liver Disease (PLD)
- Sec61γ	400		Glioblastoma
**Alternative Sec61 complex**	?		
- Sec61α2	?		
- Sec61β			
- Sec61γ			
**Chaperone network**			
- Sec63	168	ERM	Polycystic Liver Disease (PLD)
- #ERj1 (DNAJC1)	8	ERM	
- ERj3 (DNAJB11)	1,001	ERL	
- ERj4 (DNAJB9)	12	ERL	
- ERj5 (DNAJC10)	43	ERL	
- ERj6 (DNAJC3, p58^IPK^)	237	ERL	Diabetes
- ERj7 (DNAJC25)	10	ERM	
- BiP (Grp78, HSPA5)	8,253	ERL	Hemolytic Uremic Syndrome (HUS)
- Grp170 (HYOU1)	923	ERL	
- Sil1 (BAP)	149	ERL	Marinesco-Sjögren- Syndrome (MSS)
#Calnexin_palmitoylated_	7,278	ERM	
#TRAM1	26	ERM	
TRAM2	40	ERM	
PAT-10			
**#TRAP complex**		ERM	
- TRAPα (SSR1)	568		
- TRAPβ (SSR2)			
- TRAPγ (SSR3)	1,701		Congenital Disorder of Glycosylation (CDG)
- TRAPδ (SSR4)	3,212		Congenital Disorder of Glycosylation (CDG)
#RAMP4 (SERP1)		ERM	
**#Oligosaccharyltransferase**		ERM	
- RibophorinI	1,956		
- RibophorinII	527		
- OST48	273		Congenital Disorder of Glycosylation (CDG)
- N33 (Tusc3)			Congenital Disorder of Glycosylation (CDG)
- IAP			
- Dad1	464		
- OST4			
- Stt3a^*^	430		Congenital Disorder of Glycosylation (CDG)
- Stt3b^*^	150		Congenital Disorder of Glycosylation (CDG)
- Kcp2			
**Signal peptidase (SPC)**		ERM	
- SPC12	2,733		
- SPC18^*^			
- SPC21^*^			
- SPC22/23	334		
- SPC25	94		
**GPI transamidase (GPI-T)**		ERM	
- GPAA1	9		
- PIG-K	38		
- PIG-S	86		
- PIG-T	20		
- PIG-U	42		
Signal peptide peptidase		ERM	
#p34 (LRC59)	2,480	ERM	
#p180	10	ERM	
kinectin	263	ERM	

Alternative names of components/subunits are given in parentheses. We note that oligosaccharyltransferase comes in four types, comprising Stt3a or Stt3b in combination with N33 or IAP. Abundance refers to HeLa cells and is given in nM (Hein et al., 2015); C, cytosolic; ERL, ER lumenal protein; ERM, ER membrane resident;

* *, catalytically active subunit; #, ribosome associated; ?, uncharacterized in mammalian cells*.

After their targeting to the ER, precursor polypeptides with amino-terminal signal peptides or transmembrane helices associate with the Sec61 complex via their targeting peptides and trigger opening of the Sec61 channel or gating of the Sec61 channel to the open state. The latter is supported by binding of the ribosomes to the Sec61 complexes in cotranslational transport. Some precursor polypeptides require help from auxiliary components for Sec61 channel opening, such as the membrane protein complex “translocon-associated protein” (TRAP) complex or the ER lumenal Hsp70-type molecular chaperone BiP (Fons et al., 2003; Lang et al., 2012; Schäuble et al., 2012; Sommer et al., 2013). Thus, BiP and TRAP can be seen as allosteric effectors of the Sec61 channel. Subsequently, BiP and TRAP can bind to precursor polypeptides in transit through the Sec61 channel and support their partial or complete translocation by acting as molecular ratchets. This capacity was directly demonstrated for BiP by reconstitution of transport components, originally present in an ER-derived detergent extract, into proteoliposomes and their subsequent use in cell-free transport assays. Those experiments showed that inclusion of avidin into these proteoliposomes could substitute for BiP in complete and efficient translocation of precursor polypeptides, which carried biotin-modified amino acid residues, even in the case of SRP-dependent transport (Tyedmers et al., 2003). In the case of TRAP, this was suggested by cross-linking studies employing stalled precursor polypeptides and rough ER–derived membrane vesicles, i.e., rough microsomes (Conti et al., 2015). Details are given below, under the three subheadings “Structure and Dynamics of the Human Sec61 Complex during Membrane Insertion and Translocation of Polypeptides,” “Structure and Dynamics of the Human Protein Translocon during Membrane Insertion and Translocation of Polypeptides” and “Assisted Opening of the Human Sec61 Channel for Insertion and Translocation of Polypeptides.”

In many cases, membrane insertion and translocation of polypeptides in transit are accompanied by modifications, i.e., removal of signal peptides by signal peptidase, N-glycosylation by oligosaccharyltransferase (OST), or GPI anchor attachment by GPI transamidase. Simultaneously, folding and assembly of the newly imported polypeptides begins, which involves a network of molecular chaperones in the ER lumen (reviewed by Braakman and Bulleid, 2011) (“folding” in Figure 2). The central components of this chaperone network are BiP, an ATP- and Ca^2+^-dependent Hsp70-type chaperone, plus its Hsp40-type co-chaperones (ERjs or ERdj) and nucleotide exchange factors (NEFs) (reviewed by Dudek et al., 2009; Otero et al., 2010; Melnyk et al., 2014; Table 1). Furthermore, folding can involve additional chaperones, such as the glycoprotein-specific calnexin and calreticulin, and folding catalysts, i.e., protein disulfide isomerases (PDIs) and peptidylprolyl-*cis/trans*-isomerases (PPIases). Eventually, native polypeptides are passed on from the ER along the secretory pathway by vesicular transport.

The term “quality control” was coined to describe the fact that only correctly folded and assembled proteins are delivered from the ER to their functional location in the cell or outside of the cell (Ellgaard and Helenius, 2003). Mis-folded polypeptides are subjected to ER-associated protein degradation (ERAD) or a specialized form of autophagy (ER-phagy) (Figure 2). ERAD can apparently involve BiP and the Sec61 complex for the export of certain mis-folded polypeptides from the ER to the cytosol for subsequent degradation by the proteasome (reviewed by Römisch, 2005). Thus, BiP and the Sec61 complex act at the crossroads of ER protein import and ERAD. In general, however, dedicated ERAD machineries that are specialized in mis-folded ER-lumenal polypeptides or membrane proteins are involved, which are described in detail elsewhere (reviewed by Bagola et al., 2011). Most recent cryo-EM data characterized Hrd1 as the protein-conducting channel for ER export of mis-folded polypeptides (Schoebel et al., 2017). ER-phagy can involve the interaction between either one of the ER membrane proteins FAM134 and Sec62 with cytosolic protein LC3 and delivers whole ER sections for degradation within lysosomes (Khaminets et al., 2015; Fumagalli et al., 2016). We note that Sec62 is also involved in ER protein import and therefore provides a link between protein transport and quality control (Lakkaraju et al., 2012; Lang et al., 2012).

Prolonged protein mis-folding triggers the unfolded protein response (UPR); when the rescue attempt by decreased protein synthesis and increased levels of ER chaperones and ERAD components is unproductive, programmed cell death (apoptosis) is initiated (reviewed by Ma and Hendershot, 2001; Zhang and Kaufman, 2004; Figure 2). Thus, UPR and activation of the “intrinsic” pathway to apoptosis represent ER resident signal transduction pathways, which initially work to protect cells from aggregation-prone polypeptides. Ultimately they secure survival of the multicellular organism by sacrificing cells with terminal protein aggregation problems. The major players in UPR are the ER membrane proteins ATF6, IRE1, PERK, and Sig-1R. These are similar to the membrane-integrated ERjs in being transmembrane proteins and comprising a lumenal domain that can interact with BiP. In brief, these signal transduction components are inactive when BiP is bound to the lumenal domain; when BiP becomes sequestered by unfolded polypeptides, however, it is released and the signal transduction components become activated. Interestingly, IRE1 also interacts with the Sec61 complex, which adds yet another layer of UPR regulation and provides a noteworthy interconnection between ER protein import and ER stress signaling (Sundaram et al., 2017). A more detailed picture about BiP and ERjs is given in the section below titled “BiP and Its Co-factors in the Human ER, a Prolog” as well as the paragraphs concerning the assisted opening and closing of the Sec61 complex and “Novel Concept for Physiologic Roles of the Human Sec61 Channel in Cellular Calcium Homeostasis and Energy Metabolism.”

Induction of the intrinsic apoptosis pathway involves Ca^2+^ release from the ER, which may represent one potential physiological role of the passive ER Ca^2+^ leak that occurs at the level of the open Sec61 channel and is held at bay by BiP (Schäuble et al., 2012) (“Ca^2+^ signaling” in Figure 2). However, another potential role of the Sec61 complex acting in ER Ca^2+^ leakage may be related to regulation of ATP transport into the ER, which is essential for BiP activity. In any case, BiP and the Sec61 complex are also connected to intracellular Ca^2+^ signaling and cellular Ca^2+^ homeostasis. These issues are discussed below, in the sections on “Closing of the Human Sec61 Channel for Preservation of Cellular Calcium Homeostasis” and “Novel Concept for Physiologic Roles of the Human Sec61 Channel in Cellular Calcium Homeostasis and Energy Metabolism.”

We note that quality control does not occur only after membrane insertion or translocation at the level of protein folding and assembly. Proteasomes can also eliminate precursor polypeptides that were not properly targeted, which involves cytosolic protein Bag6 (Wang et al., 2011; Leznicki and High, 2012), or became stuck at the cytosolic surface of the Sec61 complex or even in transit through the Sec61 channel. The elimination option has been termed “pre-emptive quality control” by R. Hegde and involves the cytosolic ubiquitin-ligase Listerin or the ER lumenal ERj6 (Kang et al., 2006; Rutkowski et al., 2007; von der Malsburg et al., 2015). The latter option was described by M. Schuldiner as resolving translocon “clogging” and depends on the ER membrane-resident protease ZMPSTE24 (Ast et al., 2016). Interestingly, Bag6 also acts in protein targeting to the ER, and ERj6 appears to be involved in Sec61 channel closing, adding more examples to the list of pathway interconnections.

## BiP and its co-factors in the human ER, a prolog

BiP was discovered and named as an immunoglobulin heavy chain binding protein for its role in immunoglobulin assembly. It is also known as glucose-regulated protein with a mass of 78 kDa (Grp78) because it is over-produced under ER stress conditions, such as glucose starvation (Haas and Wabl, 1983). BiP is the most abundant Hsp70-type molecular chaperone in the ER lumen, reaching concentrations in the millimolar range even under non-stress conditions, and depends on ATP and Ca^2+^ for its activity (reviewed by Dudek et al., 2009; Otero et al., 2010; Melnyk et al., 2014). Another, but less abundant, member of the Hsp70 family in the ER is glucose-regulated protein with a mass of 170 kDa (Grp170). BiP and Grp170 can form a stable complex. Furthermore, various other components were found to form oligomeric complexes together with BiP, such as other chaperones, folding catalysts, and ER-resident proteins with functions in either protein transport, N-glycosylation, or cellular Ca^2+^ homeostasis (reviewed by Dudek et al., 2009).

**Figure 3:**
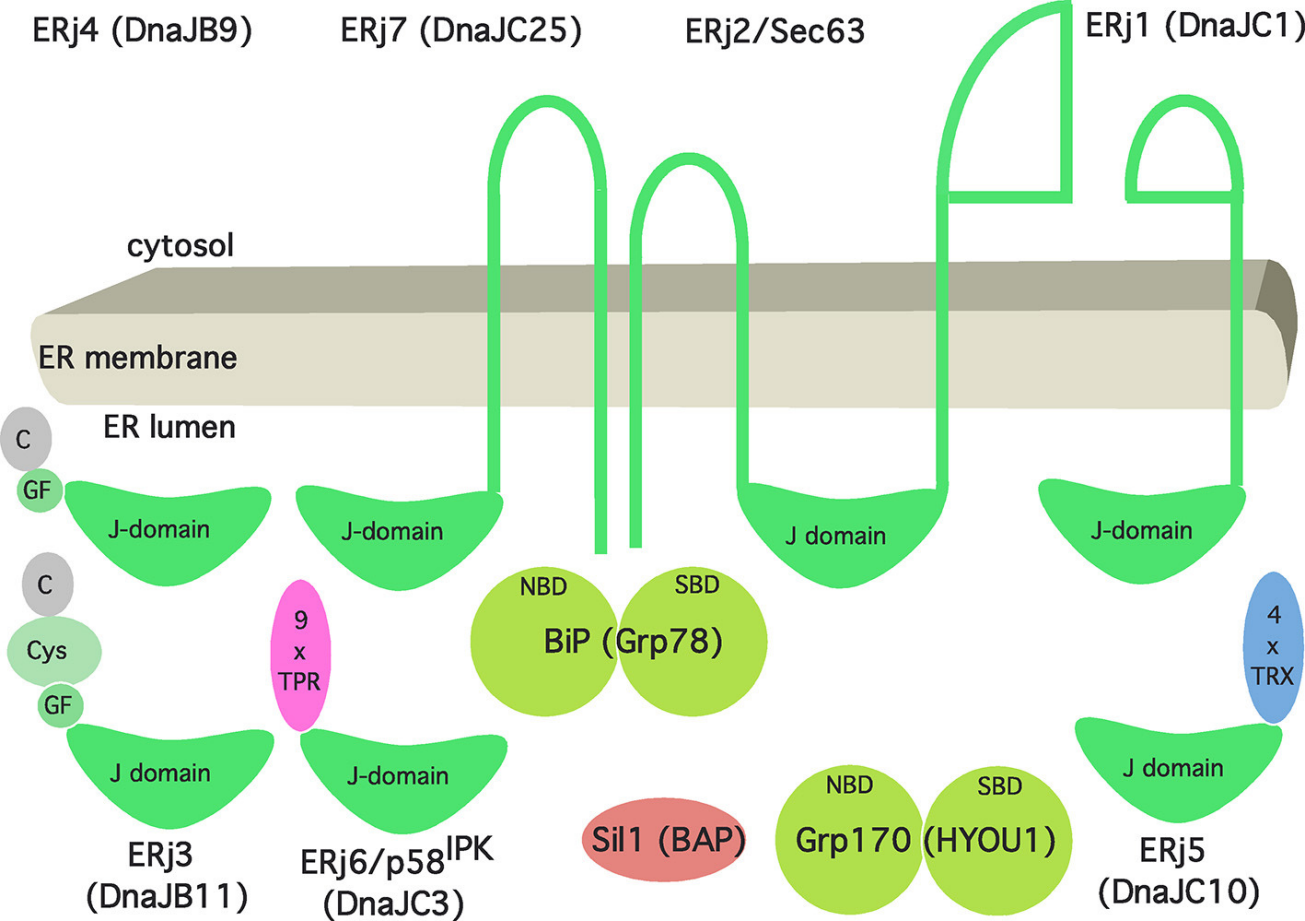
Artist's view of the Hsp70/Hsp40 chaperone network of the human ER. See text for details. The following binding characteristics (K_D_) were observed for BiP binding in the presence of ATP (in μM): ERj1, 0.12; Sec63, 5; ERj3, 3.5; ERj4, 6.07; ERj5, 0.45; ERj6, 0.59; ERj7, 1.1. The cartoon and affinities are based on Schorr et al. (2015).

Hsp70-type molecular chaperones, such as BiP, bind reversibly to substrate polypeptides via their substrate-binding domains (SBDs) (Figure 3). Typically, BiP substrates are hydrophobic oligopeptides within loosely- or un-folded polypeptides (Flynn et al., 1991; Blond-Elguindi et al., 1993). Binding of a substrate to the SBD inhibits unproductive interactions of the polypeptide and favors productive folding and assembly, which occur concomitantly with release from BiP. In addition, BiP can regulate the activities of folded polypeptides (e.g., Sec61α). This binding and release of substrates by BiP are facilitated by interaction of its SBD and its nucleotide-binding domain (NBD). NBD-conformation and BiP's ATPase cycle are modulated by different Hsp70 interaction partners (Dudek et al., 2009; Otero et al., 2010; Melnyk et al., 2014). The ATP-bound state of BiP has a low affinity for substrate polypeptides, and the ADP-bound state has a high substrate affinity. Hsp40-type co-chaperones of the ER lumen (ERjs or ERdjs or, more systematically, DNAJs) stimulate the ATPase activity of BiP and thereby favor substrate binding. NEFs of the ER lumen stimulate the exchange of ADP for ATP and thus induce substrate release.

As we have stated in more general terms before (Dudek et al., 2009), ERjs are characterized by J-domains that allow interaction with BiP via the bottom of its NBD. As of today, there are seven different ERjs present in the ER of a human cell (Figure 3), termed ERj1 through ERj7. They can be sub-classified as either ER membrane proteins or soluble ER lumenal proteins both with the characteristic lumenal J-domain. In more detail, ERjs can be classified according to the domains they have in common with the bacterial DnaJ protein (Cheetham and Caplan, 1998; Hennessy et al., 2005). “Type-I ERjs contain four domains: an amino-terminal J-domain, a glycine–phenylalanine (G/F)-rich domain, a Zn finger or cysteine repeat domain, and a carboxy-terminal SBD (ERj3). Type-II ERjs contain three domains: an amino-terminal J-domain, a G/F-rich domain, and a carboxy-terminal SBD (ERj4). Type III ERjs contain only the J-domain and, in general, have more specialized functions as compared to type I and II ERjs. Thus, ERj3 and ERj4 can bind substrate polypeptides and deliver them to BiP, i.e., facilitate polypeptide folding.” However, the four thioredoxin domains within ERj5 and the tetratricopeptide repeat domain in ERj6 (p58^IPK^) may also play a role in substrate binding. In addition, recent evidence provided further insight into the functional and regulatory role of three ERjs and how they balance Ca^2+^ flux across the ER membrane. While the pair of ERj3 and ERj6 minimizes the passive Ca^2+^ efflux across the Sec61 complex, ERj5 triggers the influx of Ca^2+^ via activation of the SERCA2 pump in a Ca^2+^ dependent manner (Schorr et al., 2015; Ushioda et al., 2016). Once Ca^2+^ levels in the ER are replenished, ERj5 is inactivated and forms oligomers. Interestingly, this circuit of Ca^2+^ flux across the ER membrane orchestrated by SERCA2 and the Sec61 complex is tightly connected to the master regulator of the UPR, BiP. On the one hand, direct binding of BiP to the lumenal loop 7 of the mammalian Sec61 complex prevents the leakage of Ca^2+^ (Schäuble et al., 2012). On the other hand, BiP, potentially in its function as classical chaperone, prevents oligomerization of ERj5 and, hence, inactivation of SERCA2 mediated Ca^2+^ influx (Ushioda et al., 2016). At a first glance, BiP seems to fine tune Ca^2+^ flux across the ER membrane. Yet, from a broader perspective, this circuitry sheds light on a potential connection between the Ca^2+^ balance of the ER and the UPR. Consequently, the passive Ca^2+^ efflux of the ER membrane might actually represent a signaling pathway reporting about protein homeostasis and folding capacity within the ER lumen.

Two NEFs are present in the ER lumen, Sil1 and Grp170 (Figure 3). Sil1 was predicted to be structurally related to cytosolic HspBP1, one of the NEFs of cytosolic Hsc70 in eukaryotes. Grp170 appears to be structurally related to Hsp110, an alternative NEF of cytosolic Hsc70 in eukaryotes. The structures of HspBP1 and Hsp110 suggested distinct interacting surfaces of their ER-lumenal equivalents with the top of BiP's NBD (reviewed by Bracher and Verghese, 2015).

## Targeting of precursor polypeptides to the Sec61 complex in the human ER membrane

A first concept for protein targeting to the ER was established by Blobel and Dobberstein (1975). In brief, an amino-terminal signal peptide in the nascent precursor polypeptide is recognized and bound by SRP in the cytosol and mediates a translational attenuation (Walter and Blobel, 1981; Halic et al., 2004, 2006; Voorhees and Hegde, 2015). The corresponding ribosome-nascent chain-SRP complex associates with the ER membrane via the heterodimeric SR, which is membrane anchored via the β-subunit (Meyer and Dobberstein, 1980; Gilmore et al., 1982; Miller et al., 1995). Interaction between SRP and SR drives the mutual hydrolysis of bound GTP and leads to release of the ribosome-nascent chain complex at the ER membrane in the vicinity of the Sec61 complex (Supplementary Video 1). Thus, in addition to its role in targeting precursor polypeptides to the ER, SRP is a molecular chaperone for nascent precursor polypeptides and an mRNA-targeting device. Interestingly, it also targets *XBP1* mRNA to the ER, where *XBP1* mRNA is cleaved by Sec61 complex associated Ire1, providing a link between ER protein import and the Ire1 branch of the UPR (Plumb et al., 2015; Kanda et al., 2016). A stalling element encoded in the 3′ region of the unspliced mRNA of XBP1 (XBP1u) leads to translational pausing after synthesis of a hydrophobic region and its emergence from the ribosomal tunnel exit (Yanagitani et al., 2011). The artifice, this mildly hydrophobic region paired with the translational arrest are allowing for unconventional SRP-mediated targeting to the Sec61 translocon, yet, avoiding efficient insertion into the ER membrane. Taking the interaction of Ire1α and the Sec61 complex into account targeting of XBP1u mRNA to the translocon allows efficient processing of XBP1u by Ire1α during ER stress conditions.

Besides SRP mediated targeting, bioinformatic analysis of the yeast secretome predicted up to 30% of all extracellular proteins being independent of SRP (Aviram and Schuldiner, 2014). Experimental identification of precursor proteins with the ability to facilitate ER targeting independent of SRP—such as GPI-anchored membrane proteins in yeast, TA membrane proteins in yeast and mammalian cells, and small presecretory proteins in the mammalian system—support the existence of alternative ER targeting machineries (Schlenstedt et al., 1990; Kutay et al., 1995; Ast et al., 2013). Accordingly, many studies determined the capacity of the ER handling a broad variety of structurally diverse precursor proteins (Stefanovic and Hegde, 2007; Schuldiner et al., 2008; Aviram et al., 2016). Their diversity is not restricted to differences in the amino acid sequence of matures domains, but equally evident in primary structure, length, hydrophobicity and location of the signal sequence itself (reviewed by von Heijne, 1985; Hegde and Bernstein, 2006). Although each of these signal sequence features has been addressed experimentally to demonstrate impact on the targeting process, the location of the targeting peptide within the precursor protein is what led to the identification of the first SRP-independent targeting route for TA membrane proteins.

TA proteins are classically defined as single spanning type 2 membrane proteins devoid of a cleavable signal sequence. Instead, TA proteins harbor a characteristic carboxy-terminally located transmembrane helix, the tail-anchor (Kutay et al., 1995; Rabu et al., 2009; Borgese and Fasana, 2011). Roughly 1% of the human genome encodes TA proteins, not all of which end up in membranes of the endo- or exocytotic pathways. TA proteins of the secretory pathway, such as the β- and γ-subunits of the Sec61 complex, Cytochrome b_5_, and many components of vesicular transport, need to be targeted and inserted into the ER membrane. Equivalent to the underlying principle of the SRP-mediated targeting, TA proteins are chaperoned in a translocation-competent fashion through the cytosol and directed to the ER membrane via an ER membrane resident receptor complex. The minimal targeting machinery for TA proteins was termed the guided entry of tail-anchored proteins (GET)-complex in yeast and TA receptor complex (TRC) in the mammalian system (Table 1). In principle, the cytosolic ATPase Trc40 with its hydrophobic binding pocket binds the TA protein, and the heterodimeric receptor complex, comprising Wrb and Caml, is required for efficient ER targeting (Stefanovic and Hegde, 2007; Vilardi et al., 2011, 2014; Yamamoto and Sakisaka, 2012). At least in yeast, orthologs of the latter two proteins are also supposed to facilitate the actual TA membrane insertion (Wang et al., 2011). Furthermore, the mammalian TA-targeting machinery involves a ribosome-associating heterotrimeric Bag6 complex (comprising Bag6, Ubl4A, and Trc35) and SGTA, which appear to act upstream of Trc40 (Leznicki et al., 2010; Mariappan et al., 2010). Interestingly, Bag6 is also involved in degradation of TA proteins, i.e., at the crossroads of targeting and quality control (Wang et al., 2011; Leznicki and High, 2012).

Although about one dozen genes encoding for yeast TA proteins were characterized as essential, knockout strains of the yeast GET machinery were viable, suggesting the existence of at least one alternative targeting route. Indeed, in 2016, a high-throughput screening approach in the lab of M. Schuldiner identified a hitherto uncharacterized targeting pathway in yeast, termed the SRP-independent (SND)-system (Aviram et al., 2016). This genetic screen used a fluorescent reporter substrate based on an obligate SRP-independent and only partially GET-dependent substrate protein. Hence, mislocalization of this reporter in any particular null mutant strain served as evidence of a targeting factor. Three novel components have been identified and characterized: Snd1, Snd2, and Snd3 (Table 1). Two hallmarks of the SND targeting pathway have been emphasized. First, similar to the SRP- and GET-targeting mechanisms, precursor substrates were targeted via the interplay of a cytosolic mediator (Snd1) and a heterodimeric receptor located at the ER membrane (Snd2, Snd3). We note that Snd1 had previously been described as a ribosome-interacting protein. Second, the SND machinery showed a preference for substrates with a central transmembrane domain. At the same time, the SND route could provide an alternative targeting pathway for substrates with a transmembrane helix at their extreme amino- or carboxy-terminus, i.e., typical SRP- or GET-dependent substrates. So far, no nucleotide requirement has been assigned to this targeting system. Sequence comparisons identified the previously characterized ER membrane protein TMEM208 as a putative human Snd2 orthologue, termed hSnd2 (Zhao et al., 2013; Aviram et al., 2016). According to experiments that combined siRNA-mediated gene silencing and protein transport into the ER of human cells in cell-free transport assays, hSnd2 appears to have the same function as its yeast counterpart (Haßdenteufel et al., 2017). So far, however, human orthologs of Snd1 and Snd3 have not been identified. Judging from the levels of SR, Wrb/Caml, and hSnd2 in HeLa cells, the impression is that the SND pathway may account for almost 30% of precursor targeting in this particular human cell (Hein et al., 2015; Table 1). Interestingly, TMEM208 was originally described as a player in ER-phagy, providing yet another link between ER protein import and protein quality control (Zhao et al., 2013).

In addition, fully synthesized precursors of small presecretory proteins in human cells were proposed to be targeted to the mammalian ER membrane in an SRP-independent fashion in several ways: (i) by their interaction with Trc40 and its putative interaction with the Trc40 receptor, (ii) by their interaction with the cytosolic protein calmodulin and its putative association with a calmodulin-binding IQ motif in the cytosolic amino-terminus of the Sec61α protein, and (iii) by direct interaction of their signal peptides with the ER membrane resident Sec62 (Shao and Hegde, 2011; Johnson et al., 2012, 2013; Lakkaraju et al., 2012). In the latter case, precursors may be chaperoned in the cytosol by Hsc70 and its Hsp40 type co-chaperones or by calmodulin, if or when the latter does not act in targeting via the IQ motif. In terms of interconnections between pathways, it is interesting to note that calmodulin was described to inhibit rather than stimulate targeting of TA proteins to the mammalian ER membrane (Haßdenteufel et al., 2011). Along the same lines, the Hsc70-interacting protein Bag1 can also deliver proteins to the proteasome, i.e., acts at the cross-roads of targeting and quality control (Alberti et al., 2003), and Sec62 can facilitate ER-phagy (Fumagalli et al., 2016).

Furthermore, the synthesis of many polypeptides is apparently initiated on ribosomes or large ribosomal subunits that are continuously attached to the ER membrane (Potter et al., 2001; Stephens et al., 2008). Therefore, direct mRNA targeting was suggested as an alternative ER-targeting mechanism, and the proteins p180 and kinectin were described as mRNA receptors in the ER membrane (Table 1). So far, there is no consensus about the possible specificity of this targeting reaction, and we are not aware of a single example of a precursor polypeptide in which mRNA targeting was a prerequisite for subsequent membrane insertion or translocation by the Sec61 complex. However, polypeptides that lack a signal peptide for ER targeting and whose synthesis was initiated on ER-bound ribosomes or large ribosomal subunits were found to be recognized by the nascent chain associated complex (NAC) (Wiedmann et al., 1994). Apparently, this interaction leads to release of the respective ribosomes from the membrane and completion of protein synthesis in the cytosol (Möeller et al., 1998; Gamerdinger et al., 2015). Thus, NAC-mediated targeting antagonism keeps the intrinsic affinity of ribosome-nascent chain complexes for the Sec61 complex in check and thereby prevents both extensive mistargeting of mitochondrial proteins to the ER and impairment of protein homeostasis in those organelles.

From a broader perspective, the emerging concept for ER protein targeting is that a molecular triage is occurring for ER-destined precursor polypeptides in the cytosol, determining the fates of nascent or fully synthesized but not-yet-folded polypeptides. It does so via a complex network of targeting signals in nascent chains and completed polypeptides and a whole variety of cytosolic factors that decode these signals. At first, these factors assist the precursors in staying in solution and remaining competent for ER targeting as well as subsequent insertion into or translocation across the ER membrane. If one of these tasks fails, the precursor is targeted to the proteasome. At later stages of protein biogenesis at the ER, this principle is repeated at the level of membrane insertion and translocation and eventually during folding and assembly.

## Structure and dynamics of the human Sec61 complex during membrane insertion and translocation of polypeptides

**Figure 4:**
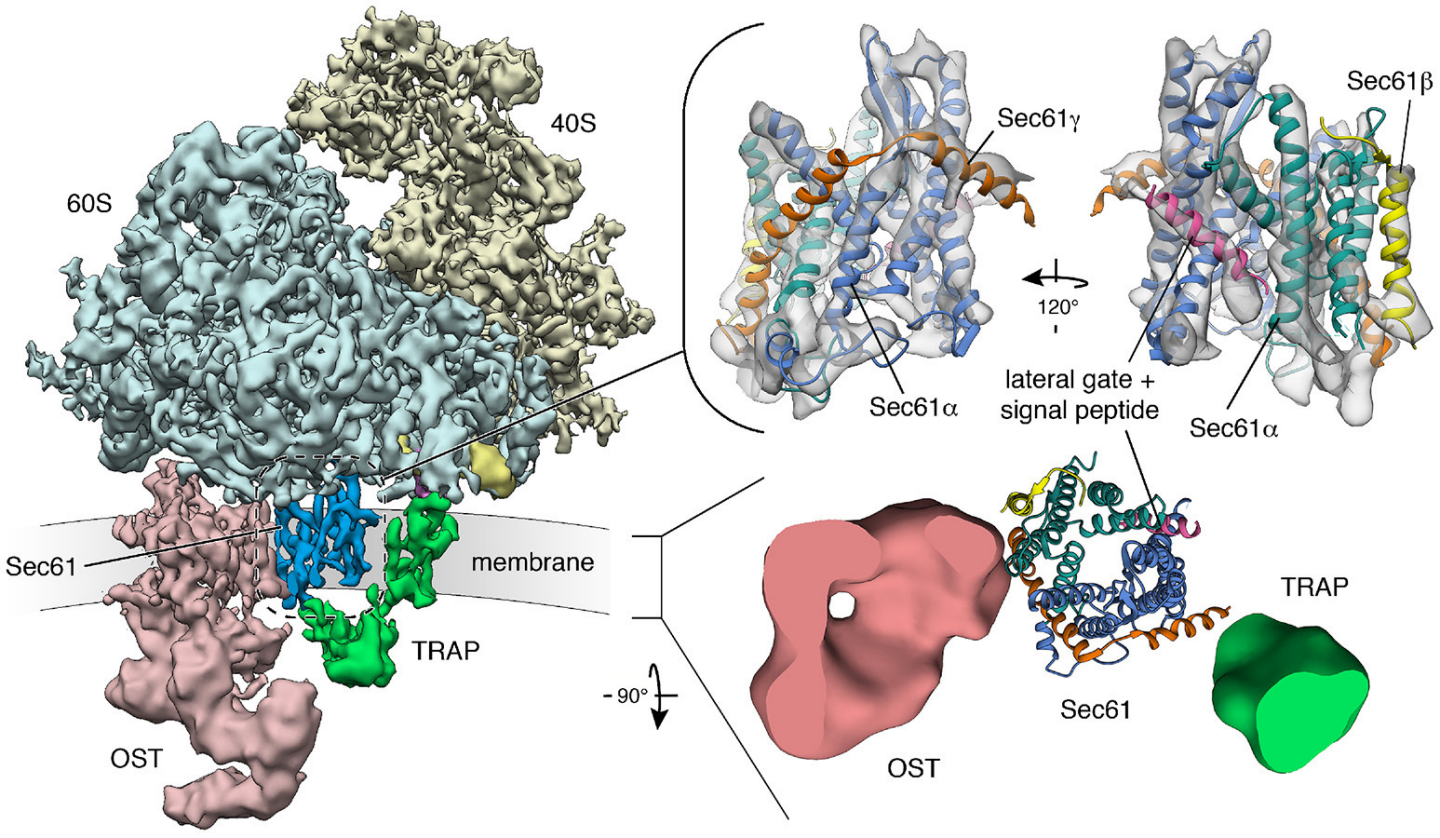
Structure and architecture of the native mammalian translocon visualized using CET. (**Left**) Overall structure of the native ribosome-translocon complex (EMD 3069) with the ribosomal subunits (40S: yellow; 60S: light blue) and the translocon components Sec61 (dark blue), TRAP (green) and OST (red) depicted. Within the 60S subunit, eL38 (purple) and the short expansion segment (bright yellow), which are contacted by the cytosolic domain of TRAPγ, are highlighted. **Right, upper panel**: Isolated density for the Sec61 complex with an atomic model of the laterally opened Sec61 complex (PDB 3jc2) superposed. The Sec61α (N-terminal: green; C-terminal half: blue), Sec61β (yellow) and Sec61γ (orange) subunits are indicated. A signal peptide (magenta) is intercalated at the lateral gate. **Right, lower panel**: Transmembrane region of the translocon with down-filtered densities for membrane-embedded segments of TRAP (green) and OST (red) depicted. Sec61 is represented by an atomic model. The ER membrane resides in the paper plane.

**Figure 5:**
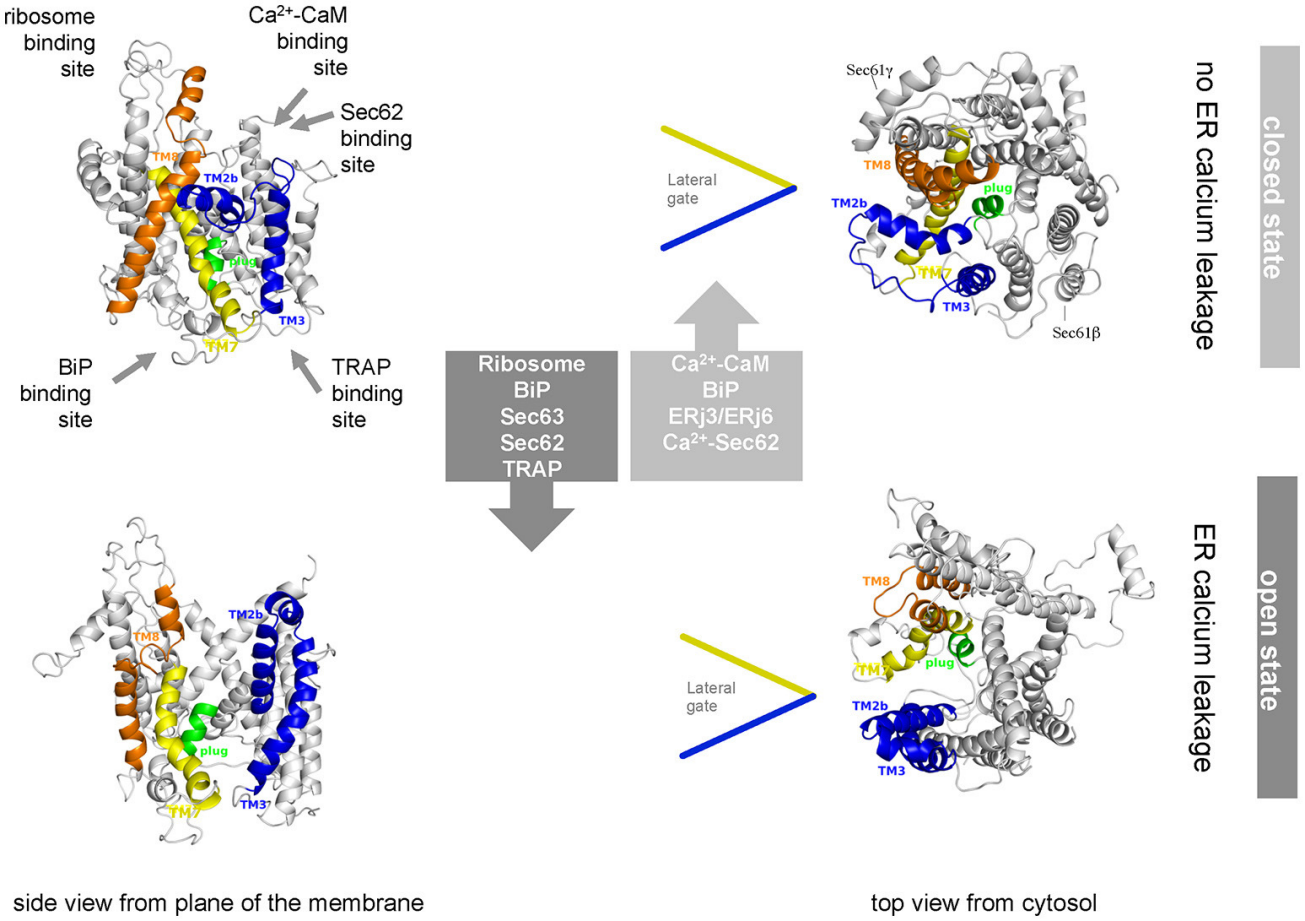
Artist's view of the dynamic equilibrium and gating mechanisms of the human Sec61 complex. Allosteric effectors of the dynamic equilibrium of the Sec61 channel and their binding sites are indicated. The cartoon is based on Dudek et al. (2015). See text for details.

**Figure 6:**
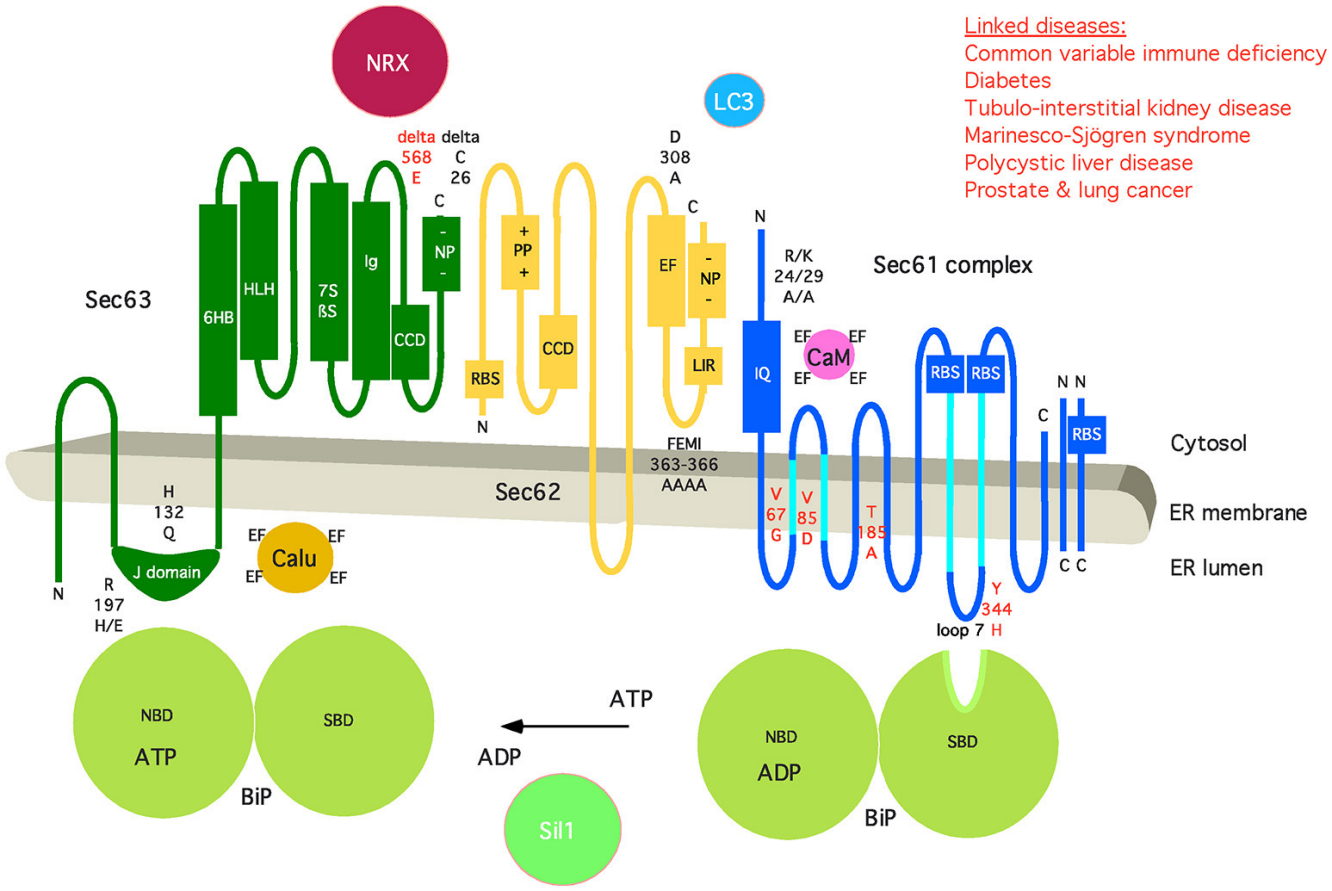
Artist's depiction of the domain organization of Sec61 complex and its auxiliary components BiP, Sec62, and Sec63. Additional interaction partners of BiP (Sil1), Sec61 (Calmodulin, CaM), Sec62 (LC3), and Sec63 (Nucleoredoxin, NRX; Calumenin, Calu) are shown. Furthermore, relevant motifs (such as IQ and LIR) and domains are indicated, as well as point mutations that disturb the respective interaction or are linked to disease (in red). CCD, coiled-coil domain; EF, EF hand; NBD, nucleotide-binding domain; NP, negatively charged patch; PP, positively charged patch; RBS, ribosome-binding site; SBD, substrate-binding domain. The following binding characteristics were observed: BiP/Sec61α K_d_ 500 μM, ATP-dependent; BiP/Sec63 K_D_ 5 μM; CaM/Sec61α K_D_ 115 nM, Ca^2+^-dependent, TFP-sensitive; Sec62/Sec61α Ca^2+^-sensitive; Sec62/LC3 K_D_ 20 μM; Sec63/NRX H_2_O_2_-dependent; Sec63/Sec62 K_D_ 5 nM. C, carboxy-terminus; N, amino-terminus. See text for details.

From a historical perspective the term “Sec” was allocated to proteins involved in protein “sec”retion and first introduced based on a yeast screen from the Schekman lab for mutants unable to efficiently secrete invertase and acid phosphatase (Novick et al., 1980; Spang, 2015). Although not among the initial 23 complementation groups, Sec61 was identified in a follow-up study also by the Schekman group (Deshaies and Schekman, 1987; Schekman, 2002). Subsequently, the structure of the hetero-trimeric Sec61 complex was first suggested by T. Rapoport and colleagues based on the X-ray crystallographic analysis of isolated archaean ortholog SecY complex (Van den Berg et al., 2004). The high sequence conservation of the SecY and Sec61 subunits indicated that their architecture and dynamics are evolutionarily conserved, which was confirmed by a number of subsequent cryo-electron microscopy (EM)-studies on detergent-solubilized or reconstituted ribosome-bound SecY or Sec61 complexes (Gogala et al., 2014; Voorhees et al., 2014). The central channel-forming subunit (Sec61α) consists of 10 transmembrane helices and is arranged in two pseudo-symmetrical amino- and carboxy-terminal halves around a central constriction which is sealed by the “pore ring,” a ring of bulky hydrophobic side chains, and a short “plug” helix (Figures 4, 5). The Sec61β and Sec61γ subunits are present on the outskirts of the Sec61 complex and contain one TA each. Strikingly, two distinct conformations of the Sec61 channel could be distinguished, which differ in the relative positioning of the amino- and carboxy-terminal Sec61α halves. These conformations either allow or don't allow lateral access of signal peptides or transmembrane helices of polypeptides in transit from the central channel toward the phospholipid bilayer through a “lateral gate” formed by transmembrane helices 2 and 7 of Sec61α (Figures 4, 5). This “lateral gate” enables insertion of nascent transmembrane helices or signal peptides emerging from the ribosome into the phospholipid bilayer. Without doubt, events at the “lateral gate” of the Sec61 complex are critical for understanding the process of protein translocation under physiological conditions, i.e., allowing transfer of a proteinaceous entity from one environment into a very different second one and simultaneously preserving the steep ER to cytosol Ca^2+^ gradient in the cell. Structural determination of programmed ribosome-Sec61 complexes implied a series of events upon arrival of a nascent precursor (Voorhees et al., 2014; Voorhees and Hegde, 2016). The idle or quiescent Sec61 complex unable to promote protein transfer is primed by binding of the ribosome to cytosolic loops 6 and 8 of Sec61α as well as the amino-terminus of Sec61γ, unveiling a hydrophobic patch in the cytosolic funnel of the engaged Sec61 complex (Figures 5, 6). This patch, in vicinity to the lateral gate, serves as an interaction site for an incoming hydrophobic signal peptide that in turn displaces helix 2 of Sec61α in order to destabilize the “lateral gate” and open the aqueous channel in the Sec61 complex for protein translocation. The cryo-EM data also demonstrated that even in cotranslational translocation, some considerable stretch of a nascent precursor polypeptide can accumulate at the interface between the ribosome and the Sec61 complex without compromising translocation (Park et al., 2014; Conti et al., 2015). Thus, elongation does not necessarily provide a driving force in translocation.

Cryoelectron tomography (CET) of translocons in native ER membrane vesicles derived from human cell lines or primary fibroblasts and even intact cells has given unprecedented insights into the architecture and dynamics of the Sec61 channel in its physiological setting and of the native translocon (Pfeffer et al., 2014, 2015, 2017; Mahamid et al., 2016). The atomic model of the solubilized ribosome-bound Sec61 complex (Voorhees et al., 2014), opened laterally by a signal peptide, was easily docked into the CET density, defining the position and conformation of Sec61 subunits in the center of the native translocon (Figure 4). Furthermore, weak helical density opposing the “lateral gate” in the CET density map confirmed the position of signal peptides, as it had been observed after detergent solubilization of ribosome-nascent chain-bound Sec61 complexes. Sec61 was found with an open “lateral gate,” possibly suggesting that Sec61 remains laterally open throughout protein translocation. At this point, the aqueous channel in the center of the complex is most likely occupied by the polypeptide chain in transit. However, computational sorting of subtomograms implied that the majority of ribosome–translocon complexes are idle and, therefore, not engaged in membrane protein insertion or protein translocation, although they were characterized by an open “lateral gate.” A possible explanation for laterally open Sec61 bound to idle ribosomes may be that even after termination of protein synthesis, signal peptides or transmembrane helices remain bound to Sec61 and keep the “lateral gate” open. In line with this view, helical density coinciding with the position of signal peptides was observed opposite of the “lateral gate” also for idle ribosome–Sec61 complexes. In this case, the aqueous channel in the center of the complex should be closed by the “pore ring” and/or the “plug” helix.

## Structure and dynamics of the human protein translocon during membrane insertion and translocation of polypeptides

As we have previously outlined (Zimmermann et al., 2011), “the first hints on participation of additional components in cotranslational protein transport came from the analysis of ribosome-associated ER membrane proteins present in detergent extracts of mammalian canine pancreatic microsomes. The term ribosome-associated membrane proteins (RAMPs) was coined for this class of membrane proteins after their solubilization in the presence of 400 mM potassium chloride (Görlich and Rapoport, 1993). By definition, the Sec61 complex is a RAMP, and so are RAMP4, TRAP and OST (Table 1). More recently, ERj1 and Sec62 were characterized as RAMPs, although their ribosome association is seen only under more physiological salt concentrations (up to 200 mM potassium chloride) and therefore may be more dynamic compared with the high-salt resistant RAMPs (Blau et al., 2005; Dudek et al., 2005; Benedix et al., 2010; Müller et al., 2010).

Additional information on the composition of the native protein transport machinery in the ER membrane came from fluorescence resonance energy transfer (FRET) experiments, which employed fluorescently labeled antibodies against transport components, permeabilized canine cells, and fluorescence microscopy.” According to this more physiological experimental strategy, Sec61α1, Sec61β, Sec62, and ERj1 are RAMPs, i.e., they are associated with ribosomes in the intact ER (Snapp et al., 2004; Benedix et al., 2010; Müller et al., 2010). Furthermore, this approach demonstrated that SR, the TRAP complex, and translocating chain-associating membrane (TRAM) protein are permanently in close proximity to Sec61 complexes. Recent cross-linking data suggested that SR and Sec62 interact with Sec61α in a mutually exclusive manner and may use the same binding site at the cytosolic amino-terminus (Jadhav et al., 2015). Therefore, it was proposed that SR can switch the Sec61 channel from Sec62- to SRP-dependent translocation.

Other experiments addressing the dynamics of the human protein translocon showed that precursors of ERj3 and prion protein depend on BiP, Sec62, and Sec63 in their ER import in cell-free transport experiments (Lang et al., 2012; Schäuble et al., 2012). Additional cross-linking experiments with stalled, radiolabeled precursor polypeptides in transit through the translocon of canine pancreatic ER membranes demonstrated that Sec62 and Sec63 only transiently associate with the Sec61 complex in a substrate-specific manner (Conti et al., 2015). Both precursor polypeptides analyzed, ERj3 and prion protein, appeared to recruit Sec62 and Sec63 to the Sec61 complex rather late in their synthesis, i.e., at precursor polypeptide chain lengths of around 150 amino acid residues. And, their signal peptides become accessible to ER-lumenal signal peptidase at chain lengths of almost 200 amino acid residues. Interestingly, a similar situation, i.e., a dynamic recruitment of Sec62 and Sec63, could be forced even for preprolactin by introducing a tightly folded zinc finger domain in the presence of Zn^2+^. As would be expected based on previous cross-linking studies with nascent preprolactin chains, preprolactin was first processed by signal peptidase at a chain length of 140 residues and found in complex with Sec61 complex, TRAP, TRAM, and OST at this stage of translocation. We conclude from these observations that in contrast to preprolactin, the two precursors of ERj3 and prion protein may contain “weak” or slowly-gating signal peptides. As a result, opening of the Sec61 channel occurred late in their synthesis, and extended sections of these two precursor polypeptides accumulated at the interface between the ribosome and the Sec61 complex triggering a rearrangement of the translocon composition to facilitate precursor translocation.

We note that a permanent association of ribosome-associated Sec61 complexes with TRAP and OST was confirmed in the recent three-dimensional (3D) reconstructions after CET of native translocons in ER membrane vesicles, derived from canine pancreas or various human cells and even intact cells (Pfeffer et al., 2014, 2015, 2016; Mahamid et al., 2016; Figure 4). Interestingly, all ribosome-associated Sec61 complexes were routinely found to be associated with TRAP, irrespective of the cellular origin of the native complexes. However, the occupancy of these Sec61/TRAP super-complexes by OST varied from one cell type to the next. While the OST occupancy was found to be around 70% in dog pancreas microsomes and microsomes isolated from several other cell types specialized in protein secretion, only 35% of translocon complexes contained OST in microsomes isolated from HeLa or HEK cells and in intact HeLa cells (Pfeffer et al., 2016). So far, our efforts to locate the position of further ones of the abovementioned translocon components have not been successful. At present, only TRAM remains a candidate for permanent and stoichiometric presence in the translocon, because it does not comprise lumenal or cytosolic domains large enough for detection using CET. Therefore, it may represent the density that is consistently found opposite of the “lateral gate” in CET of native translocons (Pfeffer et al., 2012).

**Figure 7:**
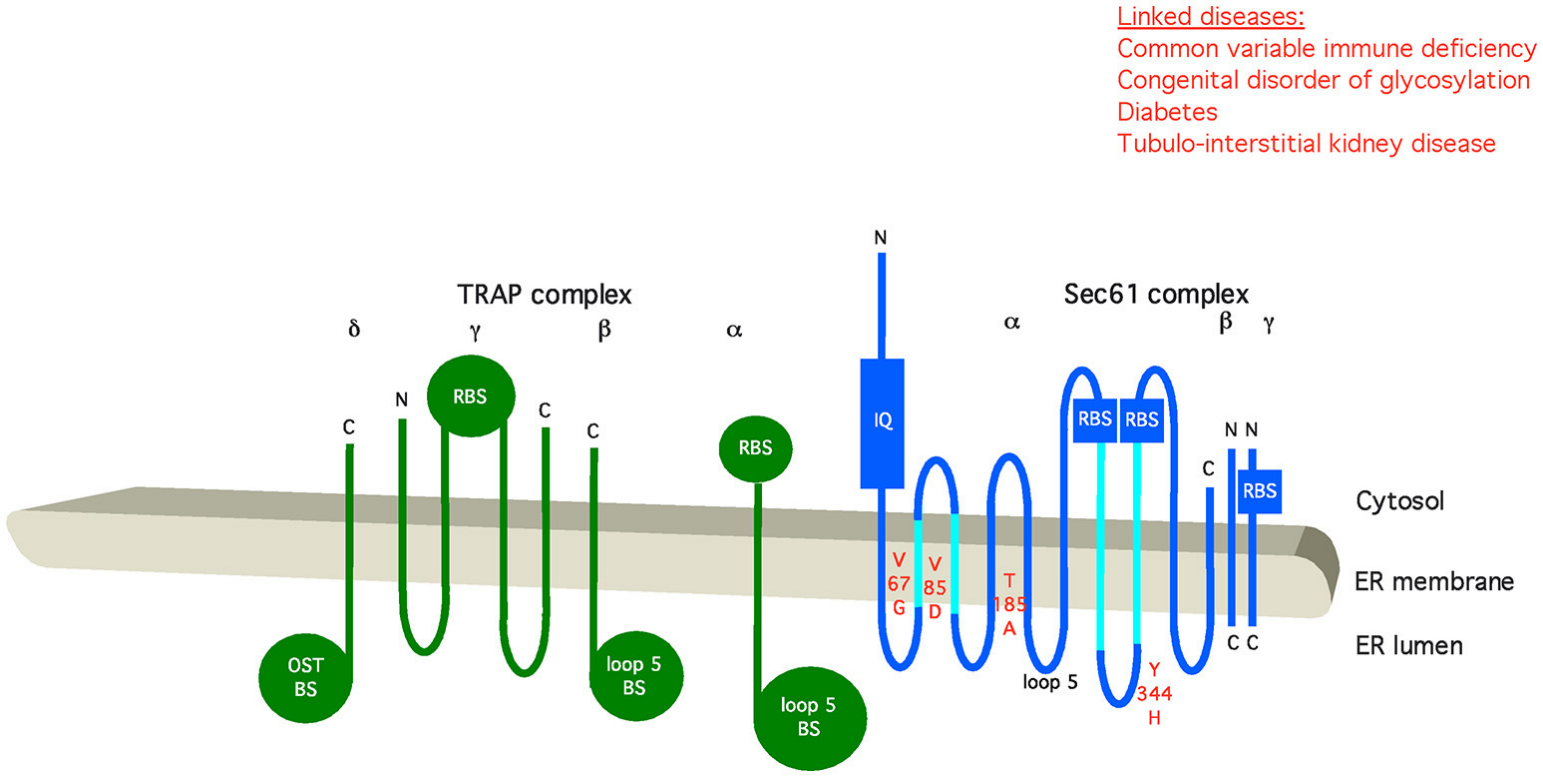
Artist's depiction of the organization of Sec61 complex and its auxiliary component TRAP. Relevant motifs (IQ) and domains are indicated, as well as point mutations that disturb the respective interaction or are linked to disease (in red). BS, binding site; OST, oligosaccharyltransferase; RBS, ribosome-binding site. C, carboxy-terminus; N, amino-terminus. See text for details.

Mammalian TRAP is a heterotetrameric membrane protein complex, with three subunits (α, β, δ) predicted to comprise one transmembrane helix plus one lumenal domain each, while TRAPγ likely comprises a bundle of four transmembrane helices plus a cytosolic domain (Hartmann et al., 1993; Bañó-Polo et al., 2017; Pfeffer et al., 2017; Figure 7). This bundle of transmembrane helices appears to be flanking both Sec61γ and the carboxy-terminal half of Sec61α (Figures 4, 7), and the cytosolic domain seems to interact with the ribosome via ribosomal protein eL38 and a short RNA expansion segment. The heterotrimeric ER-lumenal segment of TRAP reaches across the central Sec61 channel and binds to the crucial “hinge” region between the amino- and carboxy-terminal halves of Sec61α. Within the trimeric lumenal TRAP segment, the δ-subunit contacts OST (most likely ribophorin II), and the dimer formed by the lumenal domains of α- and β- subunits contacts ER lumenal loop 5 in the “hinge” region between the amino- and carboxy-terminal halves of Sec61α. In this position, the ER lumenal domain of TRAP may be able to act in a chaperone-like fashion on the conformational state of Sec61α or as a molecular ratchet on nascent precursor polypeptides in transit into the ER lumen or both, in analogy to BiP. We note that various algorithms predict a beta sandwich fold for the ER lumenal domains of TRAP's α- and β- subunits and that TRAPα was also characterized as Ca^2+^-binding protein (Wada et al., 1991).

## Assisted opening of the human Sec61 channel for membrane insertion and translocation of polypeptides

**Figure 8:**
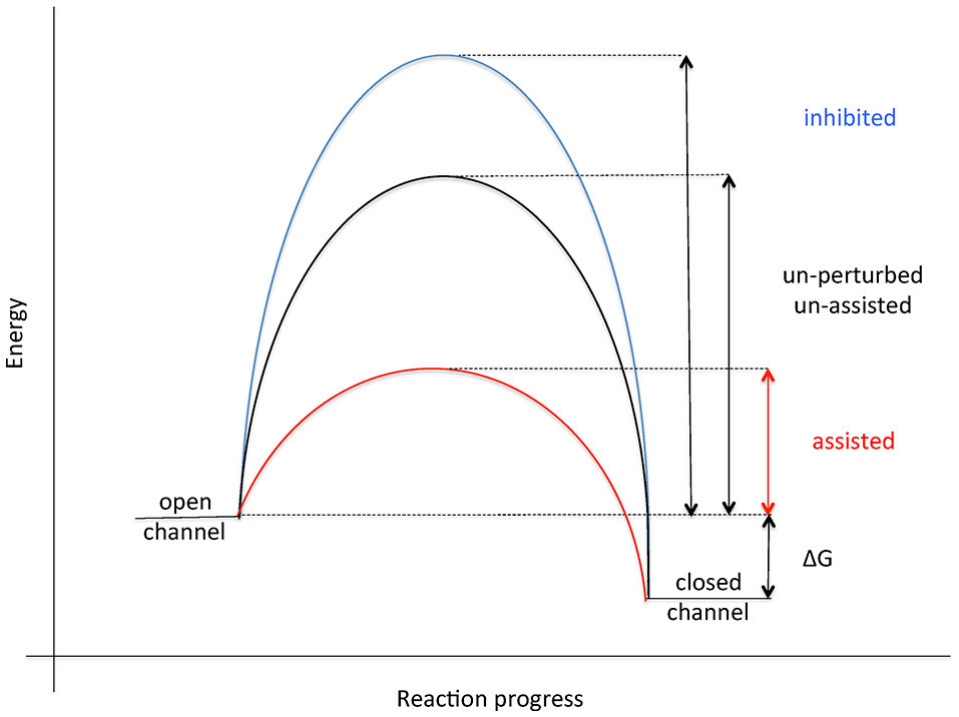
Energetics of Sec61 channel gating. See text for details.

The current view on opening of the Sec61 complex for protein translocation, i.e., channel gating from the closed to the open conformation, is that signal peptides of nascent presecretory polypeptides intercalate between the Sec61α transmembrane helices 2 and 7, displace helix 2, and open the “lateral gate” of the Sec61 complex formed by these two transmembrane helices (Van den Berg et al., 2004; Gumbart et al., 2009; Voorhees et al., 2014; Figure 4). Actually, it has been suggested that this intercalation rather than the originally proposed displacement of the “plug” helix represents the crucial reaction in the early phase of membrane insertion of translocation, i.e., the energetic barrier for Sec61 channel opening (Figure 8). Next, the nascent chain can be fully inserted into the Sec61 channel, either in “hairpin” (where the amino-terminus of the signal peptide stays in the cytosol) or “head-first” configuration (where the amino-terminus of the signal peptide reaches into the ER lumen), and initiate translocation (Devaraneni et al., 2011; Park et al., 2014; Vermeire et al., 2014). The “hairpin” insertion is considered to represent the more productive mode whereas a “head-first” insertion has to be followed by a reversal of orientation (termed “flip turn”) to allow the sequence downstream of the signal peptide to enter the ER lumen. The latter may be considered a second energetically unfavorable reaction, typically requiring help from components, which can lower the energetic barrier for the “flip turn” (Figure 8). The idea is that some amino-terminal signal peptides or transmembrane helices may be “strong” or quickly-gating enough to trigger Sec61 channel opening quickly on their own, particularly after the ribosome has already primed the channel. However, precursor polypeptides with “weak” signal peptides appear to involve auxiliary components in Sec61 channel opening in order to facilitate insertion of precursor polypeptides into the Sec61 complex (Table 1). Alternatively, the auxiliary components may support the abovementioned “flip turn” in case of an original “head-first” insertion. Based on *in vitro* experiments the concept emerged that TRAP and BiP facilitate Sec61 channel opening in a substrate specific manner. In particular, precursor polypeptides with “weak” signal peptides or transmembrane helices are affected (Fons et al., 2003; Schäuble et al., 2012; Figure 5; Supplementary Video 1). Based on only a small set of model precursor polypeptides, the distinguishing factor that determines the requirement for BiP and its membrane bound co-chaperone Sec63 was suggested to be a short and rather apolar signal peptide, eventually to support it in displacing helix 2 of Sec61α on its own account. The TRAP complex was observed in *in vitro* transport studies to stimulate translocation of specific proteins, such as the prion protein. Recent studies in intact cells have suggested that TRAP might also affect the topology of transmembrane helices that do not promote a specific initial orientation of membrane protein precursors in the membrane (Sommer et al., 2013). As noted before (Haßdenteufel et al., 2014), several additional proteins in the mammalian ER membrane can be considered as auxiliary translocon components, most notably TRAM (Voigt et al., 1996; Hegde et al., 1998). In the case of TRAM, signal peptides of precursors with long amino-terminal as well as long hydrophobic core regions showed a low TRAM dependence in *in vitro* experiments. Interestingly, there is a second TRAM in mammalian cells, termed TRAM2, which can invert the topology of transmembrane helices that do not promote a specific initial orientation in the membrane (Chen et al., 2016).

We suggest that certain features of signal peptides may extend the “dwell” time or “sampling” of signal peptides on the cytosolic surface of the Sec61 channel and that BiP and TRAP can overcome this by facilitating Sec61 channel gating on the lumenal side (Zhang and Miller, 2012; Van Lehn et al., 2015). This raises the exciting possibility that BiP and TRAP have overlapping specificities, i.e., that there is also redundancy in this reaction, as discussed above for the targeting reaction. Another interesting and equally open question is what features make a signal peptide or transmembrane helix “weak” or “strong” for Sec61 channel opening and if it is really only these topogenic sequences that determine this “weakness” or “strength.” Some features have already been mentioned above but were determined using only small sets of model proteins. However, our own unpublished work suggests that special features downstream of the signal peptides can also play a distinct role, which may be particularly relevant in cotranslational translocation when a considerable stretch of a nascent precursor polypeptide accumulates at the interface between ribosome and Sec61 complex, i.e., prior to Sec61 channel opening (see above) and in posttranslational translocation (S. Haßdenteufel, personal communication). This is reminiscent of the effects of downstream sequences in the integration of transmembrane helices into the membrane (Junne and Spiess, 2017). Interestingly, yeast Sec62 and mammalian TRAP were found to affect the topology of transmembrane helices that do not promote a specific initial orientation of membrane protein precursors in the membrane (Reithinger et al., 2013; Sommer et al., 2013).

In the case of BiP, it has been suggested that the minihelix within loop 7 of Sec61α plays a role in gating of the Sec61 complex from closed to open and that BiP binding to this minihelix may be required for only some precursor polypeptides (Figures 5, 6). “Thus, by providing binding energy, the ribosome and BiP may be able to ‘pull’ transmembrane helix 7 from opposite ends to facilitate channel opening (Figure 8; Schäuble et al., 2012). We find this hypothesis attractive because loop 7 connects transmembrane helices 7 and 8 and is thus close enough to the “lateral gate” to influence gate movements.” Thus, BiP together with Sec63 protein represents an allosteric effector of the Sec61 complex for channel opening. This view was supported by the observations that the murine diabetes-linked mutation of tyrosine 344 to histidine within loop 7 destroys the BiP binding site and, when introduced into HeLa cells, prevents *in vitro* transport of BiP-dependent, i.e., slowly-gating precursor polypeptides.

As stated above, the dimer formed by the lumenal domains of the α- and β- subunits of TRAP contacts ER lumenal loop 5 in the “hinge” region between the amino- and carboxy-terminal halves of Sec61α (Pfeffer et al., 2017; Figures 4, 7). Thereby, it may act as an alternative allosteric effector of Sec61 channel and thus may facilitate opening of the Sec61 channel to allow initiation of protein translocation and topogenesis of membrane proteins, in analogy to the action of BiP on loop 7. Therefore, the question arises of how TRAP may signal the presence of a signal peptide requiring help in Sec61 channel gating to the ER lumenal TRAP domains (see above).

## Closing of the human Sec61 channel for preservation of cellular calcium homeostasis

As discussed before (Zimmermann, 2016), “the mammalian ER is also a central player in cellular calcium homeostasis (Figures 1, 2). It represents the major Ca^2+^ storage organelle in nucleated mammalian cells and allows controlled release of Ca^2+^ from the ER upon hormone stimulation of a resting cell, e.g., via IP3 receptor (Berridge, 2002; Clapham, 2007). Subsequently, Ca^2+^ is pumped back into the ER by sarcoplasmic/ER Ca^2+^ ATPase (SERCA) to re-establish the steep ER to cytosol Ca^2+^ gradient (Wuytack et al., 2002). This gradient is also constantly challenged by passive Ca^2+^ efflux from the ER, so SERCA has the additional task of counteracting this Ca^2+^ leakage. In addition, Ca^2+^ is taken up by mitochondria. In the course of the last 10 years, several proteins were linked to ER Ca^2+^ leakage, including the Sec61 channel (Lomax et al., 2002; Van Coppenolle et al., 2004; Erdmann et al., 2011). Other candidate proteins that were identified acting as putative Ca^2+^ permeable leak channel at the ER membrane are presenilin1 (Tu et al., 2006), Bcl2 (Chami et al., 2004), pannexin1 (Vanden Abeele et al., 2006), TRPC1 (Berbey et al., 2009), CALHM1 (Gallego-Sandín et al., 2011), and a truncated SERCA1 isoform (Chami et al., 2001, 2008). Some of those candidate proteins, however, were ruled out as passive Ca^2+^ leak channels allowing the efflux of Ca^2+^ from the ER observed in all nucleated cells. For example, presenilin was shown to have a stimulatory effect on IP3 receptors (Cheung et al., 2008, 2010), i.e., triggering a rather direct Ca^2+^ release from the ER. In addition, mature presenilin is predominantly located in the plasma and Golgi membrane (https://www.proteinatlas.org). Similarly, mature Bcl2, pannexin1, and TRPC1 are not present at the ER membrane to act as ubiquitous Ca^2+^ leak channel and their property as leak channel was addressed upon overexpression. Calcium homeostasis modulator 1 (CALHM1) increased Ca^2+^ efflux from the ER and reduced activity of SERCA (Gallego-Sandín et al., 2011), but the restricted and low expression of CALHM1 in tissues of the brain, kidney, bladder and immune cells render it an unlikely candidate as ubiquitous Ca^2+^ leak channel (https://www.proteinatlas.org). The proposed reverse Ca^2+^ flux through the SERCA pump of myocytes could represent yet another source of Ca^2+^ efflux from the ER (Shannon et al., 2000). Interestingly, a short splice variant of SERCA1 (S1T) found in different human tissues reduces ER Ca^2+^ loading via increased passive Ca^2+^ efflux from the ER and reduces activity of SERCA1 and SERCA2. S1T is induced during ER stress, homodimerizes and elevates ER Ca^2+^ depletion for induction of apoptosis, thus rendering S1T a specialized Ca^2+^ leak channel under stress conditions (Chami et al., 2001, 2008). However, the ubiquitously expressed, ER resident Sec61 complex with its pore forming subunit represents an ideal candidate as omnipresent passive Ca^2+^ leak channel. A genome-wide RNAi screen in Drosophila S2 cells identified Sec61α (but none of the aforementioned candidates) as component reducing Ca^2+^ release-activated Ca^2+^ channel activity (Zhang et al., 2006). Though, with such a highly abundant Ca^2+^ leak channel it is imperative to prevent excessive ER Ca^2−^-efflux and disturbance of the Ca^2+^-gradient across the ER membrane. Therefore, Sec61 channel gating has to be tightly controlled as described below (Figure 5).

Single-channel recordings from planar lipid bilayers characterized the Sec61 complex as a highly dynamic aqueous channel with a main calcium conductance of 165 ± 10 pS and a subconductance state of 733 ± 16 pS allowing a rough estimation about the opening diameter of the pore from 5 to 7 Å for the main conductance and 12–14 Å for the subconductance state. The Sec61 complex is transiently opened by signal peptides within precursor polypeptides and is permeable to Ca^2+^ at the end of protein translocation (Simon et al., 1989; Wirth et al., 2003; Erdmann et al., 2011; Lang et al., 2011). The same experimental strategy showed that the Sec61 channel closes either spontaneously or as induced by binding of BiP or Ca^2+^-calmodulin (Erdmann et al., 2011; Schäuble et al., 2012). The fact that BiP is involved in closing the Sec61 channel was confirmed at the cellular level by combination of siRNA-mediated gene silencing or pharmacological manipulation and live cell Ca^2+^ imaging (Schäuble et al., 2012). In addition, cytosolic Ca^2+^-calmodulin was shown under similar conditions to contribute to Sec61 channel closing via an unrelated mechanism once Ca^2+^ has started to leak from the ER (Erdmann et al., 2011). During the last 5 years, additional siRNA-mediated gene silencing and live cell Ca^2+^ imaging experiments characterized the pair of ERj 3 and 6 as co-chaperones of BiP as well as Ca^2+^-Sec62 as a co-factor of calmodulin in Sec61 channel closure (Linxweiler et al., 2013; Schorr et al., 2015). Furthermore, the binding sites of BiP, Ca^2+^-calmodulin, and Ca^2+^-Sec62 were identified as the abovementioned di-tyrosine motif–containing mini-helix within ER lumenal loop 7 of the Sec61 α-subunit and an IQ motif in the cytosolic amino-terminus of the same subunit, respectively (Figure 5). Furthermore, the respective affinities of these interactions were determined by surface plasmon resonance spectroscopy and found to be physiologically relevant (Figure 6).

The following scenario for gating of the Sec61 channel has emerged from these studies (reviewed by Zimmermann et al., 2011; Dudek et al., 2015; Pfeffer et al., 2016; Zimmermann, 2016; Figure 5). As described above, binding of a precursor polypeptide to the closed Sec61 complex triggers channel opening, either on its own or facilitated by binding of the allosteric modulator of the Sec61 channel, BiP (Schäuble et al., 2012; Figure 5). Here, Sec63 acts as a BiP co-chaperone (Lang et al., 2012). After completion of protein translocation, i.e., in the absence of any bound precursor polypeptide, the channel closes on its own, or BiP facilitates efficient gating of the Sec61 channel to the closed state (Schäuble et al., 2012). At this stage, ERj3 and ERj6 are BiP co-chaperones, possibly acting in the form of a heterodimeric complex (Schorr et al., 2015). The idea is that binding of BiP to loop 7 of Sec61α provides energy for shifting the dynamic equilibrium of the Sec61 channel to the closed state. The idea that such a mechanism may indeed be at work came from single-channel recordings where Fab fragments directed against loop 7 could substitute for BiP in channel closing (Schorr et al., 2015). In case of inefficient channel closure in intact cells, Ca^2+^ starts to leak from the ER into the cytosol and binds calmodulin, and Ca^2+^-calmodulin is recruited to the IQ motif in the Sec61 α-subunit (Erdmann et al., 2011; Figures 5, 6). Once again, the involved binding energy may favor channel closure. Binding of Ca^2+^-calmodulin is supported by Sec62, which may have bound Ca^2+^ because of a predicted EF hand within its cytosolic carboxy-terminal end (Linxweiler et al., 2013). Next, the Sec61 channel is closed, and Ca^2+^ leakage subsides. SERCA pumps Ca^2+^ back into the ER, calmodulin and Sec62 return to the Ca^2+^-free forms, and the next protein translocation cycle can be initiated. The crucial open question is if or when the Ca^2+^ permeability of the open Sec61 channel and its elaborate control mechanisms play a physiological role.

As previously outlined (Linxweiler et al., 2017), “an additional function beyond ER protein import and Ca^2+^ homeostasis was recently found for the Sec62 protein and represents yet another example of pathway overlaps (Fumagalli et al., 2016). Sec62 also plays a crucial role in the recovery of eukaryotic cells from conditions of ER stress. In the course of UPR, the level of several ER lumenal chaperones such as BiP is markedly increased (Ma and Hendershot, 2001; Zhang and Kaufman, 2004). If the cell can cope with ER stress conditions, the expanded ER as well as the high amount of ER lumenal chaperones have to be returned to a physiological level. Therefore, small vesicles derived from the ER membrane fuse with phagophores to build autophagosomes (ER-autophagy) (Figure 6). For this purpose, Sec62 bears a LIR motif at its carboxy-terminus that functions as a receptor for phagophore-bound LC3. Thus, Sec62 plays an important, Sec61- and Sec63-independent role during recovery from ER stress.” A similar mechanism may be involved in ER-phagy when mis-folded polypeptides overwhelm the ERAD machinery and whole ER sections have to be sacrificed to protect the cell. We suggest that phosphorylation of the negative patch in the carboxy-terminus of Sec63 and/or Ca^2+^ binding to the EF hand in the carboxy-terminus of Sec62 may trigger dissociation of Sec62 from its interaction partners (Ampofo et al., 2013; Linxweiler et al., 2013).

## Novel concept for physiologic roles of the human Sec61 channel in cellular calcium homeostasis and energy metabolism

As stated above, the Ca^2+^ permeability of the open Sec61 channel may be involved in the intrinsic pathway to apoptosis, i.e., when cells have to be sacrificed to protect a multicellular organism from terminal protein aggregation problems. We suggest that under conditions of severe and prolonged protein mis-folding and aggregation even after UPR induction, BiP is terminally sequestered by mis-folded and aggregating polypeptides. As described, this sequestration will eventually lead to continuous Ca^2+^ leakage from the ER via open Sec61 channels (Figure 2). In the long run, the effect may contribute to increased cytosolic Ca^2+^ levels, which are typically involved in induction of apoptosis. We expect that such a scenario may be particularly relevant for secretory cells, such as the β cells of the pancreas or plasma cells of the immune system. Therefore, these two cell types are particularly sensitive to mutations in the *SEC61A1* gene (see below).

To fulfill its central role in protein biogenesis, the ER of all nucleated human cells contains the ATP dependent chaperone BiP in millimolar concentration and, thus, depends on a constant supply of ATP. So far, only for the plant *Arabidopsis thaliana* an ER-resident membrane ATP carrier has been described (Leroch et al., 2008). Thus, the question remains of how ATP gets into the human ER. Recent work has established a set of hallmarks for this nucleotide transport (Vishnu et al., 2013). There appears to be a regulatory circuit for maintenance of ATP supply of the human ER that involves ER-lumenal and cytosolic Ca^2+^, the elusive ADP/ATP carrier, and cytosolic AMP–activated protein kinase (AMPK) (Figure 2). Decreasing ATP levels in the ER leads to decreasing ER Ca^2+^- and increasing cytosolic Ca^2+^ levels, where the former activates the ER membrane–resident ADP/ATP carrier and the latter stimulates ADP phosphorylation in cytosol and mitochondria. Based on our observations on the Ca^2+^ permeability of the open Sec61 channel and its limitation by BiP, we propose that the above-described regulatory circuit for maintenance of ATP supply of the human ER also involves the Sec61 channel in the ER membrane and the ER lumenal BiP. According to this novel concept, decreased ATP levels in the ER should cause lower BiP activity, which in turn causes ER Ca^2+^ leakage via the Sec61 channel, in analogy to the situation where BiP is sequestered by mis-folded polypeptides. Next, decreasing ER lumenal Ca^2+^ activates the ER membrane–resident ADP/ATP carrier, and increasing cytosolic Ca^2+^ stimulates ADP phosphorylation in cytosol and mitochondria. Subsequently, ATP levels in the ER recover, BiP binds to the Sec61 channel and stops Ca^2+^ from leaking into the cytosol. This scenario may also serve as a framework for envisioning how breakdown of energy metabolism can cause apoptosis, e.g., when plasma cells are at the end of their lifespan. In this case, accumulation of mis-folded immunoglobulin polypeptide chains may further aggravate the situation (Kourtis and Tavernarakis, 2011).

## Sec61-channelopathies and therapeutic strategies

**Figure 9:**
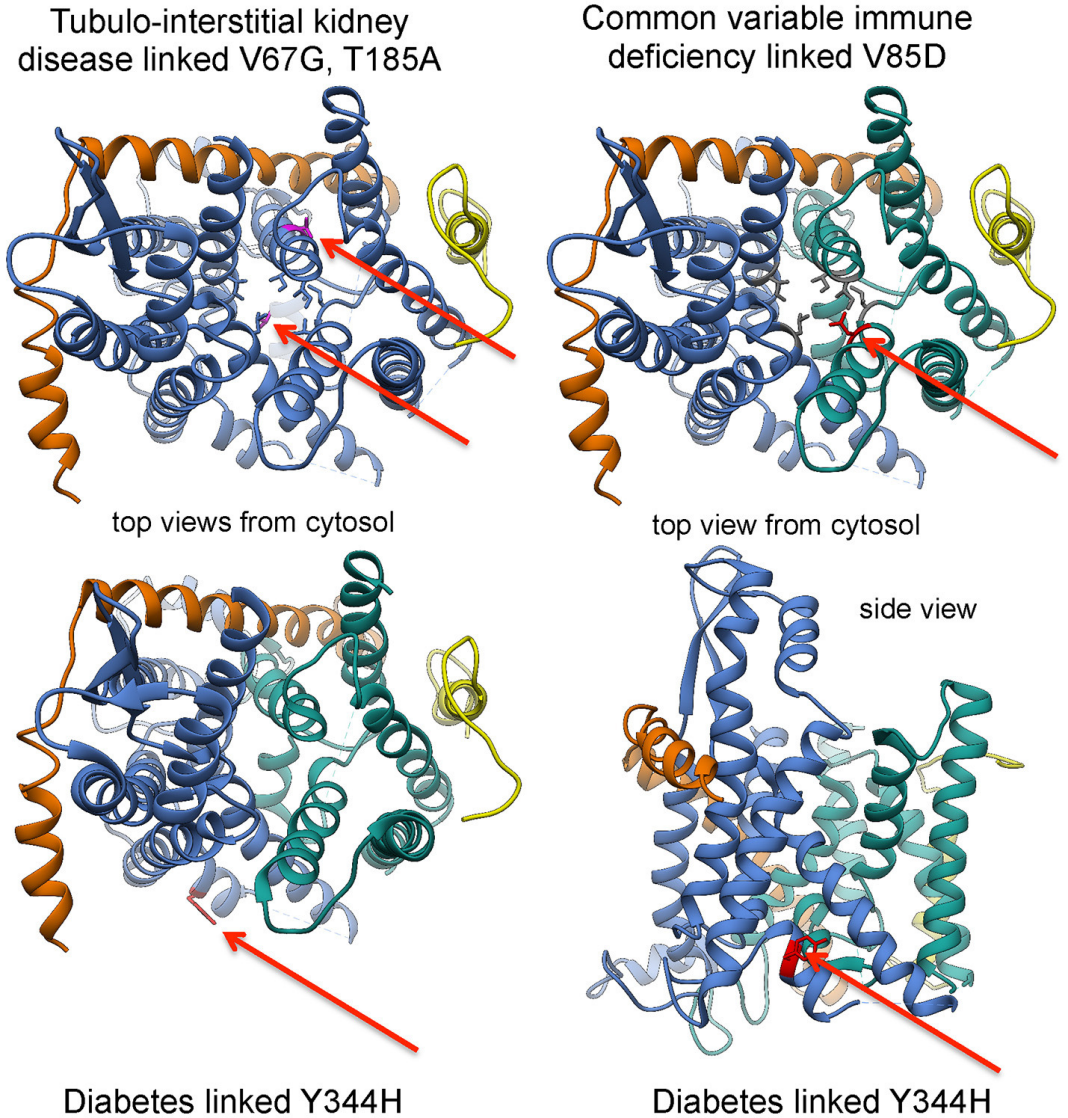
Position of disease-linked mutations in 3D reconstructions of the Sec61 complex. See text for details.

“In light of this elaborate system of Sec61 channel gating, it did not come as a surprise that various diseases were linked to components of the protein translocation machinery (Zimmermann, 2016).” The term Sec61-channelopathies was coined for the family of inherited or tumor-related diseases that either directly affect Sec61 subunits or are linked to components involved in Sec61 channel gating (Haßdenteufel et al., 2014; Linxweiler et al., 2017; Table 1; Figures 5, 9). Mutations in the gene coding for the Sec61 α-subunit can cause diabetes in the mouse (Lloyd et al., 2010), and common variable immune deficiency (CVID) and tubulo-interstitial kidney disease with anemia in humans (Bolar et al., 2016; Schubert et al., 2017). Loss-of-function mutations in genes coding for Sec63 and ERj6, respectively, were linked to autosomal dominant polycystic liver disease and diabetes in both humans and mice (Davila et al., 2004; Ladiges et al., 2005; Fedeles et al., 2011; Synofzik et al., 2014). Furthermore, polycystic liver disease can be caused by heterozygous mutation of the *SEC61B* gene (Besse et al., 2017), and proteolytic inactivation of BiP by the bacterial subtilase cytotoxin SubAB causes the devastating hemolytic uremic syndrome (Paton et al., 2006). Overproduction of components of the protein translocation machinery is associated with cancers of prostate, lung, head, and neck (Sec62) and with glioblastoma (Sec61γ) (Lu et al., 2009; Greiner et al., 2011; Linxweiler et al., 2012, 2013; Bochen et al., 2017). In addition, several diseases have been linked to subunits of OST (reviewed by Mohorko et al., 2011) and Sil1 (Senderek et al., 2005; Zhao et al., 2005; Roos et al., 2014), respectively, and appear to affect N-glycosylation of newly synthesized polypeptides and protein folding, respectively, rather than Sec61 channel gating (Table 1). As described before (Zimmermann, 2016), “the human diseases associated with mutations in OST and Sil1 are congenital disorders of glycosylation (CDG) Type I and the neurodegenerative Marinesco-Sjögren Syndrome, respectively. We note that CDG can also result from loss-of-function mutations in genes coding for different subunits of TRAP (Losfeld et al., 2014; Pfeffer et al., 2017; Table 1).

In the case of diabetes, loss of ERj6 function and homozygous *SEC61A1* mutation, respectively, were suggested to be caused by inefficient gating of Sec61 channels to the closed state with sustained ER Ca^2+^ leakage and, eventually, apoptosis of secretory cells, such as pancreatic ß cells (Schäuble et al., 2012; Schorr et al., 2015). The former is in agreement with ERj6 being involved in gating of the Sec61 channel to the closed state. The latter was explained by the observation that the diabetes-linked mutation of the *SEC61A1* tyrosine 344 to histidine affects the di-tyrosine motif-containing mini-helix of the Sec61 α-subunit, i.e., the BiP-binding site (Schäuble et al., 2012; Figures 5, 9).” As a consequence, the mutated Sec61 channel cannot be efficiently gated by BiP and thus becomes permeable for Ca^2+^. A similarly permeable Sec61 channel may exist in case of CVID, where a heterozygous mutation of the *SEC61A1* gene (resulting in the substitution of valine 85 by aspartate) introduces a polar amino acid side chain into the typically non-polar “pore ring” of the Sec61 channel (Schubert et al., 2017). This view was supported by the observation that the CVID-linked mutation, when introduced into HeLa cells, leads to permeable Sec61 channels, which may have a dominant-negative effect on the cells. Therefore, the lifespan of plasma cells may be shortened in the patients. Alternatively, the disease phenotype may be caused by haploinsufficiency. The functional consequences are less clear in the case of tubulo-interstitial kidney disease, where two mutations in the *SEC61A1* gene have been reported (resulting in the substitution of valine 67 by glycine and of threonine 185 by valine), which are located in the “plug” domain and transmembrane helix 5, respectively (Bolar et al., 2016). In all diseases that are related to the ubiquitously expressed *SEC61A1* gene, the crucial question is why a particular mutation affects only a single cell type.

In the case of polycystic liver disease, reduction or loss of Sec63 function appears to cause a precursor polypeptide-specific defect in ER protein import, which results in the absence of certain plasma membrane proteins, such as polycystin 1, involved in planar cell polarity (Davila et al., 2004; Fedeles et al., 2011). This association is consistent with the idea that Sec63 is involved in gating of the Sec61 channel to the open state. In addition, the interaction of Sec63 with cytosolic nucleoredoxin may be relevant for the disease phenotype (Müller et al., 2011). Apparently, the loss-of-function mutation of the *SEC61B* allele also causes a precursor-specific ER protein import defect. Again, the open question is why only a certain cell type, in this case cholangiocytes, is affected by the partial or complete loss of function.

It appears that excessively efficient closing of the Sec61 channel can also lead to disease (Linxweiler et al., 2012, 2013). Amplification and/or over-expression of the *SEC62* gene (also termed *TLOC1*) were linked to various cancers and appear to be associated with poor prognosis. *SEC62* over-expression was found to result in elevated migratory potential and increased stress tolerance of the respective tumor cells, i.e., two “hallmarks” of cancer cells with a connection to cellular Ca^2+^ homeostasis. Furthermore, the *SEC62* gene has been characterized as a “tumor driver gene” (Hagerstrand et al., 2013). The two cancer hallmarks of *SEC62* over-expressing tumor cells can be overcome by siRNA-mediated gene silencing (Linxweiler et al., 2013). Based on our data on the role of Sec62 in Sec61 channel gating, we asked whether the effect of *SEC62* silencing on *SEC62* over-expressing tumor cells can be phenocopied by drugs. We reasoned that if Ca^2+^-calmodulin secures efficient Sec61 channel closure in cooperation with Ca^2+^-Sec62, calmodulin antagonists should mimick the effect of *SEC62* silencing on *SEC62* over-expressing tumor cells; indeed, this is what we found. One particular calmodulin antagonist of interest is trifluoperazine, since it has previously been in clinical use for depressive patients. Thus, we are currently addressing in murine tumor models if proliferation of *SEC62* over-expressing tumor cells can be inhibited by a combinatorial treatment that includes trifluoperazine and a SERCA inhibitor. We note that SERCA-targeting prodrugs are currently being evaluated in clinical trials (Mahalingam et al., 2016).

## Sec61 channel inhibitors, an epilog

In the course of the last 10 years, several small molecule inhibitors of the Sec61 channel have been discovered which, in analogy to mutations of the *SEC61A1* gene, affect ER protein import in a precursor-specific or non-selective manner. The first-described and precursor-selective class of such inhibitors were the cyclic heptadepsipeptides, i.e., CAM749 and cotransins (such as CT8) (Besemer et al., 2005; Garrison et al., 2005; MacKinnon et al., 2014). Subsequently, the structurally unrelated compounds apratoxin A and mycolactone were characterized as Sec61 effectors and shown to have selective (mycolactone) or non-selective (apratoxin A) effects on ER protein import by interaction with the channel (Liu et al., 2009; Hall et al., 2014; Baron et al., 2016; McKenna et al., 2016, 2017; Paatero et al., 2016). The model to explain a precursor-specific inhibitory effect suggests that certain signal peptides and transmembrane helices can either bypass or displace the drugs during their initial insertion into the Sec61 channel. Thus, both possibilities may, at least in some way, reiterate the above discussion of “weak” and “strong” signal peptides (see above): the bound small molecules may increase the energy barrier involved in opening of the Sec61 channel for protein translocation and precursors with “strong” signal peptides may overcome the barrier anyhow (Figure 8). Alternatively, selective inhibitors may occupy binding sites within the Sec61 channel, which are irrelevant for some signal peptides. Therefore, the exact mode of action of these compounds is an important open question. Furthermore, it will be interesting to address the questions of whether or not the selectivity of some of the small molecules correlates with the dependence of some precursors on certain auxiliary components in gating of the channel and if and how the inhibitory compounds affect cellular Ca^2+^ homeostasis.

Intriguingly, heptadepsipeptides are considered for the treatment of multiple myeloma, which is very much in line with the observation in CVID patients that physiological levels of functional Sec61 channels are essential for plasma cell viability. Mycolactone appears to be a good candidate to follow that same path.

## Concluding remarks

The mammalian Sec61 complex forms a dynamic and precursor gated channel, which can provide an aqueous path for polypeptides into the ER lumen and is regulated by various allosteric effectors. When the aqueous path is open, it can apparently also provide a channel for efflux of calcium ions from the ER lumen into the cytosol. We suggest that this feature is linked to the regulation of ATP import into the ER and the initiation of the intrinsic pathway to apoptosis, respectively. To us, the most pressing open questions concern (i) the structure of the native Sec61 complex in the ribosome-free state, (ii) the positioning of other transport and processing components within the native translocon, (iii) the rules of engagement of the allosteric effectors of the Sec61 channel plus their molecular mechanisms. The latter will undoubtedly also pave the way for a detailed understanding of the pathomechanisms which are involved in Sec61 channelopathies. Another burning question is the nature of the elusive ATP carrier(s) of the mammalian ER membrane.

## Author contributions

SP and FF contributed 3D reconstructions after CET. PL and VH performed molecular modelings. SL and RZ wrote the first draft of the manuscript, which was contributed to by all authors.

### Conflict of interest statement

The authors declare that the research was conducted in the absence of any commercial or financial relationships that could be construed as a potential conflict of interest.

## Supplementary material

Supplementary Video 1 Artist's view of SRP/SR, BiP and Sec63 mediated transport of presecretory proteins via the Sec61 channel into the ammalian ER. J, J-domain of Sec63 recruits BiP to the Sec61 channel for channel opening and to incoming precursor polypeptides for ratcheting, respectively. Signal peptidase (SPase) cleaves the signal peptide from the incoming precursor polypeptide. See text for details.
