# Supplementary figures and images for: An Update on Sec61 Channel Functions, Mechanisms, and Related Diseases
Source: Front Physiol. 2017 Nov 1;8:887. doi: 10.3389/fphys.2017.00887 (PMC5672155; doi:10.3389/fphys.2017.00887)

## Slide 1
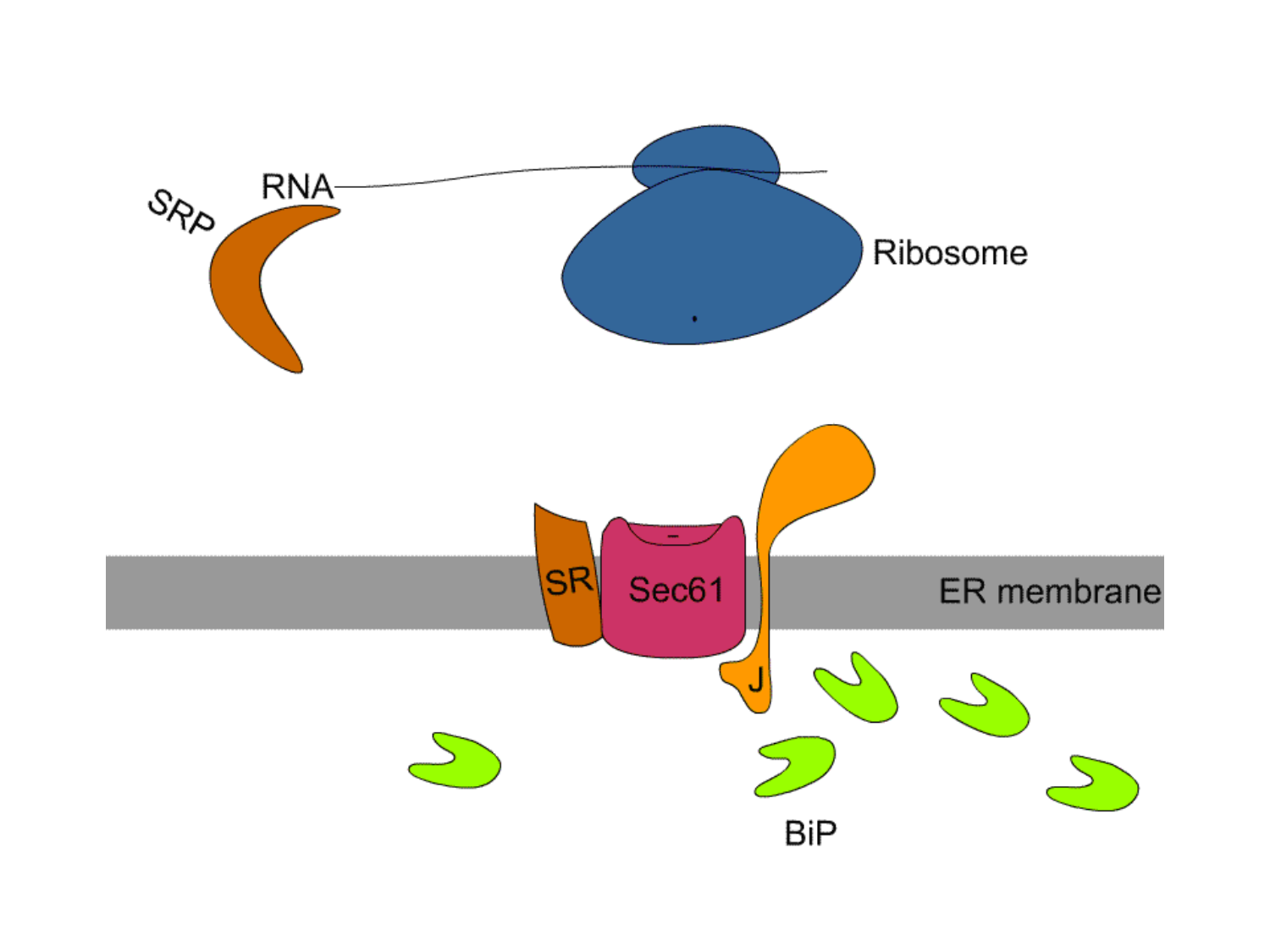

Supplement: Supplementary Video 1 — Artist's view of SRP/SR, BiP and Sec63 mediated transport of presecretory proteins via the Sec61 channel into the ammalian ER. J, J-domain of Sec63 recruits BiP to the Sec61 channel for channel opening and to incoming precursor polypeptides for ratcheting, respectively. Signal peptidase (SPase) cleaves the signal peptide from the incoming precursor polypeptide. See text for details. [file Presentation1.PPTX]
